# MiR-192, miR-200c and miR-17 are fibroblast-mediated inhibitors of colorectal cancer invasion

**DOI:** 10.18632/oncotarget.26263

**Published:** 2018-10-30

**Authors:** Volker Ast, Theresa Kordaß, Marcus Oswald, Amol Kolte, David Eisel, Wolfram Osen, Stefan B. Eichmüller, Alexander Berndt, Rainer König

**Affiliations:** ^1^ Integrated Research and Treatment Center, Center for Sepsis Control and Care, Jena University Hospital, 07747 Jena, Germany; ^2^ Network Modeling, Leibniz Institute for Natural Product Research and Infection Biology, Hans Knöll Institute Jena, 07745 Jena, Germany; ^3^ GMP & T Cell Therapy Unit, German Cancer Research Center, 69120 Heidelberg, Germany; ^4^ Institute of Forensic Medicine, Section Pathology, Jena University Hospital, 07747 Jena, Germany

**Keywords:** mixed-integer linear programming, piecewise linear regression, extracellular matrix, tumor-associated fibroblast, tumor cell invasion

## Abstract

Colorectal cancer remains a leading cause of cancer-related death worldwide. A previous transcriptomics based study characterized molecular subgroups of which the stromal subgroup was associated with the worst clinical outcome. Micro-RNAs (miRNAs) are well-known regulators of gene expression and can follow a non-linear repression mechanism. We set up a model combining piecewise linear and linear regression and applied this combined regression model to a comprehensive colon adenocarcinoma dataset. We identified miRNAs involved in regulating characteristic gene sets, particularly extracellular matrix remodeling in the stromal subgroup. Comparison of expression data from separated (epithelial) cancer cells and stroma cells or fibroblasts associate these regulatory interactions with infiltrating stromal or tumor-associated fibroblasts. MiR-200c, miR-17 and miR-192 were identified as the most promising candidates regulating genes crucial for extracellular matrix remodeling. We validated our computational findings by *in vitro* assays. Enforced expression of either miR-200c, miR-17 or miR-192 in untransformed human colon fibroblasts down-regulated 85% of all predicted target genes. Expressing these miRNAs singly or in combination in human colon fibroblasts co-cultured with colon cancer cells considerably reduced cancer cell invasion validating these miRNAs as cancer cell infiltration suppressors in tumor associated fibroblasts.

## INTRODUCTION

Colorectal cancer is a severe and lethal disease, accounting for almost 10% of all cancer-related deaths worldwide (in 2014, see [1]). The advent of deep-sequencing technologies have provided valuable insight into the genomic and transcriptomic landscape of large colorectal sample cohorts [2], allowing the definition of distinct molecular subgroups [3]. However, to develop targeted, subgroup-specific treatment strategies, a mechanistic understanding of gene expression regulation is required. Since the discovery of microRNAs (miRNA), the knowledge and mechanistic understanding of gene regulation by miRNAs has expanded considerably. Several studies elucidated that miRNAs cause gene dysregulation and, by this, acting as tumor suppressors or oncomiRs [4]. Aberrantly expressed miRNAs have been shown to actively participate in tumor initiation, development, progression and invasion in multiple human cancer types [5], which makes them attractive targets for new therapeutic strategies. Particularly, miRNAs suit as prognostic markers and may serve as attractive candidates for targeted therapy of colorectal cancer [6]. Functional assays using miRNA transfection [7] and clinical trials show promising results to support miRNA-based treatment of specific cancer types, e.g. miR-34a targeting oncogenes and genes involved in tumor immune evasion [8]. Typically, miRNAs are expressed in a spatiotemporal-specific pattern. Their regulating effect depends on the cell type, tissue, RNA-induced silencing complex (RISC) availability, binding site abundance and seed sequence complementarity. It was observed that more abundant miRNAs can repress their targets to a higher degree [9]. However, luciferase target assays in *Drosophila* revealed that even miRNAs expressed at similar levels exhibited quite different repression effects [9]. In other studies, the authors investigated the repression of targets based on different miRNA dosages and concluded that only highly abundant miRNAs can effectively influence the expression of their target genes [10], suggesting a non-linear behavior. To address these observations of a threshold-dependent, non-linear regulation of target genes by miRNAs, we implemented a piecewise linear model to predict miRNA – target gene regulation using gene and miRNA expression profiles. This flexible approach approximates a non-linear behavior while still benefiting from the advantages of linear approaches such as robustness and low computation intensity. We explored miRNAs and their target gene regulation using a colon adenocarcinoma dataset [2] form The Cancer Genome Atlas (TCGA). We identified miR-192, miR-200c and miR-17 as regulators of genes involved in remodeling the extracellular matrix, in particular in the stromal subgroup of colorectal cancer. Observing transcription profiles of cancer samples sorted into stromal and tumor cells, we found this regulatory mechanism to happen in tumor-associated fibroblasts in the tumor microenvironment. This hypothesis was validated experimentally by (1) distinctive down-regulation of 85% of the predicted target genes after transfection of the identified miRNAs singly or in combination in fibroblasts, and (2) reduced invasion of colorectal cancer cells co-cultured with transfected fibroblasts employing Boyden-chamber assays.

## RESULTS

### Predicting miRNA target genes with a combined regression model outperforms predictions of linear regression models

To identify miRNA targets using miRNA and gene expression profiles from the same patients, typically, a linear regression model is set up which aims to estimate the expression of a certain target gene by the expression of one or multiple potential miRNAs taken from miRNA – target gene prediction tools or databases (see e.g. [11]). As stated above, gene regulation by miRNAs often shows a non-linear, threshold dependent behavior. Therefore, we extended the concept of linear regression models by implementing piecewise linear models (details of the mathematical realization are given in Supplementary 1.1). As a reference method, we established a standard linear regression model similar as in [12] (details, see Supplementary 1.2). We tested both methods on comprehensive sets of gene and miRNA expression profiles of two cancer entities taken from The Cancer Genome Atlas, i.e. of colon and prostate adenocarcinoma. The performance of our method (piecewise linear) and the standard method (linear regression) was evaluated by comparing the lists of predicted target genes with lists of genes being significantly down-regulated after transfection of the corresponding miRNAs in colon (or prostate) cancer cells. For this, we used publicly available miRNA transfection experiments (see Supplementary 1.3). In both datasets, the piecewise linear model outperformed the linear model in the majority of the transfection experiments, reflecting the non-linear gene regulation by miRNAs. Combining the results from both models considerably improved the target gene predictions (results in Supplementary 2.1, Supplementary 2.2 and Supplementary Table 7). In the following, we focus on the analysis of colon adenocarcinomas, and, due to its superiority, we use only the predictions from the combined regression model to identify target genes for miRNAs.

### The combined regression model identifies miRNAs and functional gene sets specific for molecular colorectal cancer subgroups

**Figure 1:**
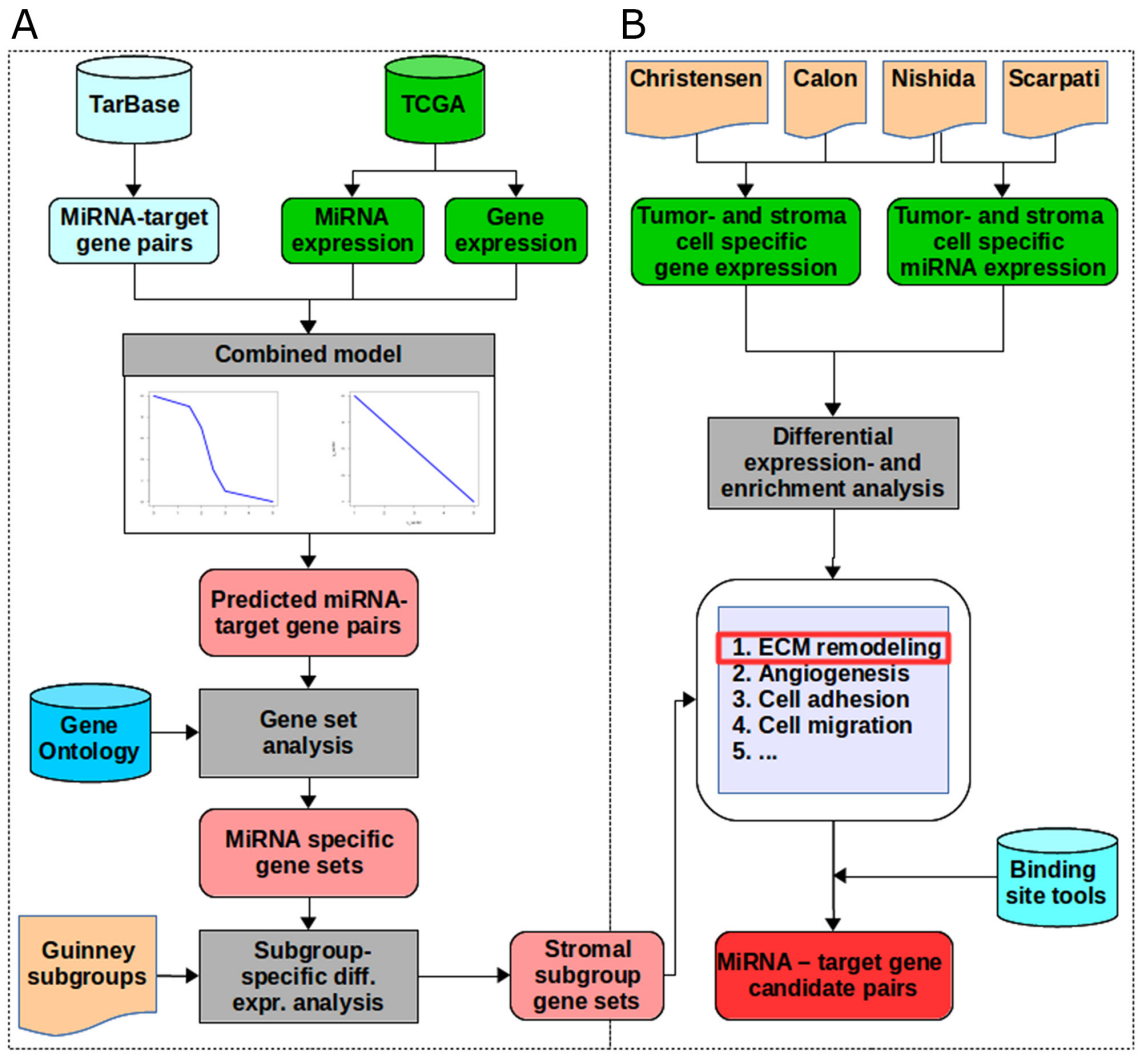
The workflow **(A)** We extracted miRNA - target gene pairs from TarBase and miRNA- and gene expression data for a colon adenocarcinoma dataset from TCGA as input for the combined model consisting of the linear and the piecewise linear model. Out of these we selected all target genes with a good prediction performance. For each miRNA, we performed gene set enrichment analysis on the target genes predicted by the combined model using the gene set definitions from Gene Ontology. By integrating colorectal cancer subgroup definitions from Guinney *et al.*, we tested for differential expression of miRNAs and genes of each subgroup *versus* all other subgroups. For each subgroup, we selected pairs with opposed expression direction (miRNA up-regulated and target genes down-regulated or *vice versa*). This resulted in subgroup-specific miRNAs and their enriched regulated gene sets. The stromal subgroup was selected for further analysis. **(B)** Among the enriched gene sets in the stromal subgroup we identified extracellular matrix remodeling (ECM) as the most prominent gene set. MiRNA and gene expression data of cells being sorted into tumor- and stroma origin were extracted from four different studies. Differential expression, enrichment analysis and integration of binding site predictions identified 39 miRNA - target gene pairs involved in ECM remodeling.

By applying the combined regression model described above, we identified a total of 10,620 miRNA - target gene pairs predicted to be regulated by 310 different miRNAs. To identify functional processes regulated by a certain miRNA, we performed gene set enrichment analysis on the predicted target genes for each miRNA. Enriched gene sets were grouped into 18 broader categories (see Supplementary 1.4 for details). To further specify miRNAs and miRNA regulated processes, we investigated their potential regulation for molecular colorectal cancer subgroups defined by Guinney *et al.* [3]. We determined differentially expressed miRNAs and genes in each subgroup and selected miRNA - target gene pairs from the enriched gene sets with opposed expression (miRNA up-regulated and target genes down-regulated or *vice versa)*, focusing on the mostly observed inhibitory effect of miRNAs. The workflow is depicted in Figure 1A.

**Figure 2:**
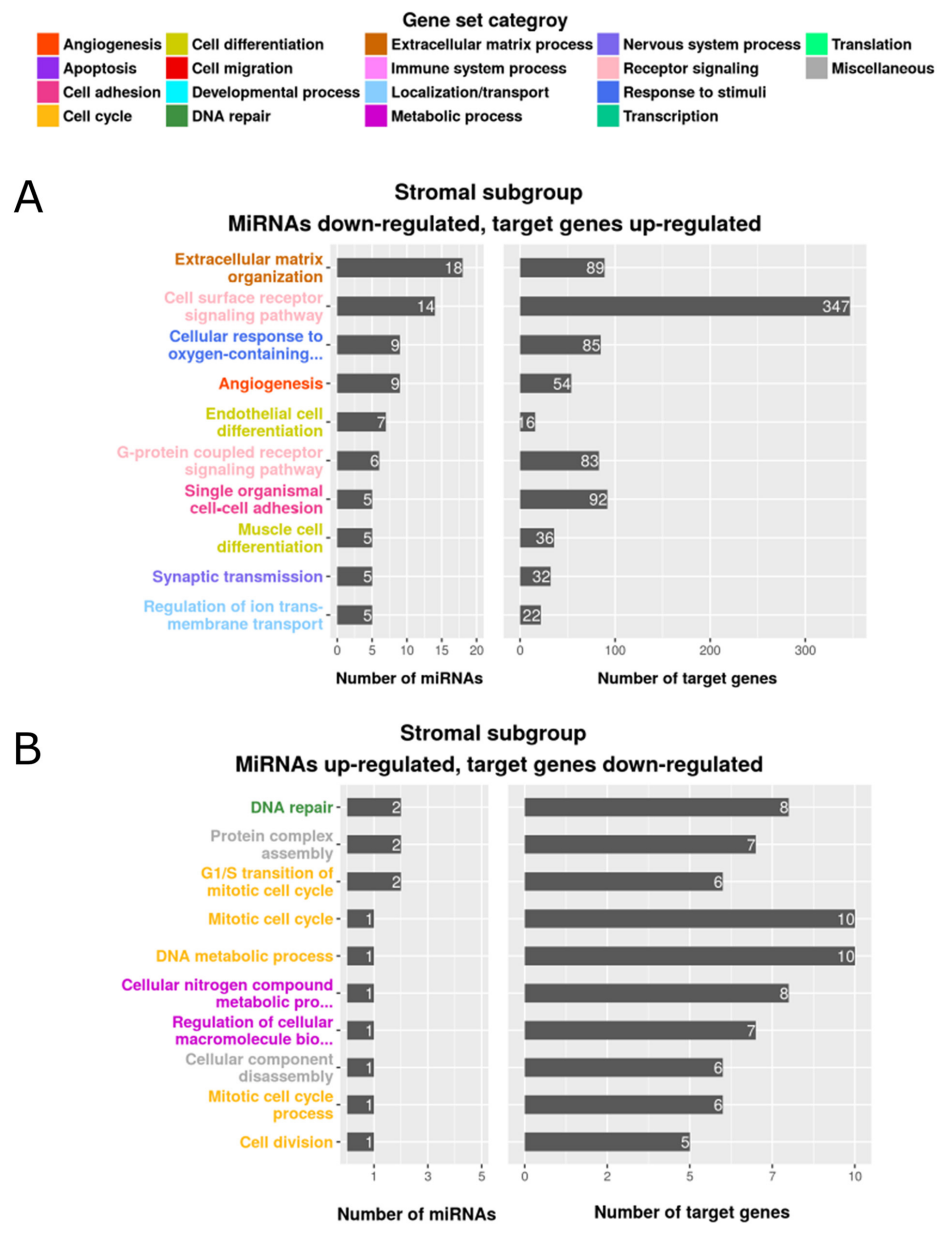
Enriched gene sets of differentially expressed genes and miRNAs in the stromal subgroup The top 10 gene sets are listed according to the number of regulating miRNAs (left) and the number of enriched target genes (right). The color indicates the assigned gene set category, **(A)** miRNAs down-regulated, target genes up-regulated, **(B)** miRNAs up-regulated, target genes down-regulated.

In the MSI immune tumor subgroup, 6 of the predicted miRNAs were down-regulated and associated with 43 enriched, up-regulated gene sets. Among the down-regulated miRNAs, miR-335 and miR-362 were enriched in gene sets related to the immune system, including *inflammatory response*, *adaptive immune response* and *T-cell differentiation*. Only 2 up-regulated miRNAs (miR-155 and miR-223) were associated with 2 down-regulated gene sets (*fatty acid oxidation* and *negative regulation of transcription from RNA polymerase II promoter*) in the MSI immune tumor subgroup. Supplementary Figure 2A-2B depicts these results and Supplementary Table 8 lists all of the identified gene sets, together with their categories. The canonical tumor subgroup had 28 up-regulated miRNAs which were associated with 169 enriched gene sets containing down-regulated target genes (Supplementary Figure 2C-D and Supplementary Table 8). A member of the miR-17∼92 cluster, miR-17, was up-regulated and associated with target genes in the functional gene sets *apoptosis*, *cell differentiation*, *cell activation* and *angiogenesis*. This is in line with Guinney et al. [3] who reported up-regulation of miR-17 and other miR-17∼92 family members in the canonical tumor subgroup. In contrast to MSI immune subgroup tumors, miR-335 and miR-362 were up-regulated and many of their target genes (miR-335: n=100, miR-362: n=12) encoding proteins involved in immune functions were down-regulated in the canonical tumor subgroup. Only two miRNAs (miR-615, miR-132) were down-regulated, corresponding with 5 enriched, up-regulated gene sets in canonical tumors. Although we identified 9 up-regulated miRNAs associated with 25 enriched gene sets in the metabolic tumor subgroup, only 2 gene sets, *carbohydrate derivative biosynthetic process* and *positive regulation of cellular catabolic process*, were associated with metabolism (Supplementary Figure 2E and Supplementary Table 8). From the 6 down-regulated miRNAs identified by Guinney et al. [3] our analysis only confirmed let-7e to be down-regulated in the metabolic tumor subgroup. However, GSEA analysis detected no enriched functional gene sets for the down-regulated miRNAs in this tumor subgroup. We identified the majority of differentially expressed miRNAs associated with functional gene sets in the stromal tumor subgroup. To note, as our data show the mesenchymal subgroup defined by Guinney *et al.* [3] to be enriched with tumor-associated stromal cells, we use the term “stromal subgroup”. These gene sets included a total of 86 down-regulated miRNAs that were associated with up-regulated target genes belonging to 289 enriched gene sets and 14 up-regulated miRNAs targeting down-regulated genes in 29 gene sets (Figure 2 and Supplementary Table 8). Target genes associated with cell cycle and DNA repair processes were down-regulated in stromal subgroup tumors and corresponded to 11 up-regulated miRNAs. We confirmed all 5 miRNAs (miR-141, miR-200a, miR-200b, miR-200c and miR-429) determined to be down-regulated by Guinney *et al.* [3], and identified further down-regulated miRNAs as potential regulators of gene sets associated with angiogenesis, cell migration and endothelial and muscle cell differentiation, including miR-17 and members of the miR-29b, miR-16 and miR-7 families. The most prominently regulated functional gene sets in the stromal tumor subgroup were *extracellular matrix organization* (18 miRNAs, including miR-192 and miR-200c, associated with 89 target genes) and *extracellular matrix disassembly* (5 miRNAs, including miR-17, associated with 19 target genes). Supplementary Table 9 summarizes miRNAs, target genes and their specific role concerning extracellular matrix synthesis, organization, maintenance and signaling. The functional grouping of the target genes is briefly described in Supplementary 1.5. The key process of extracellular matrix remodeling is central for tumor cell migration, invasion and metastasis and may at least partially underlie the poor clinical course of patients with stromal subgroup tumors. For this reason, we focused our further investigation on the regulatory role of miRNAs in extracellular matrix remodeling in the stromal subgroup tumors. Our approach using a combination of piecewise linear and linear model predicted miRNA target genes that could be linked to functional contexts and identified miRNAs as potential regulators of subgroup-specific gene sets in colorectal cancer.

### Tumor-associated fibroblasts substantially impact the phenotype of stromal subgroup tumors

**Figure 3:**
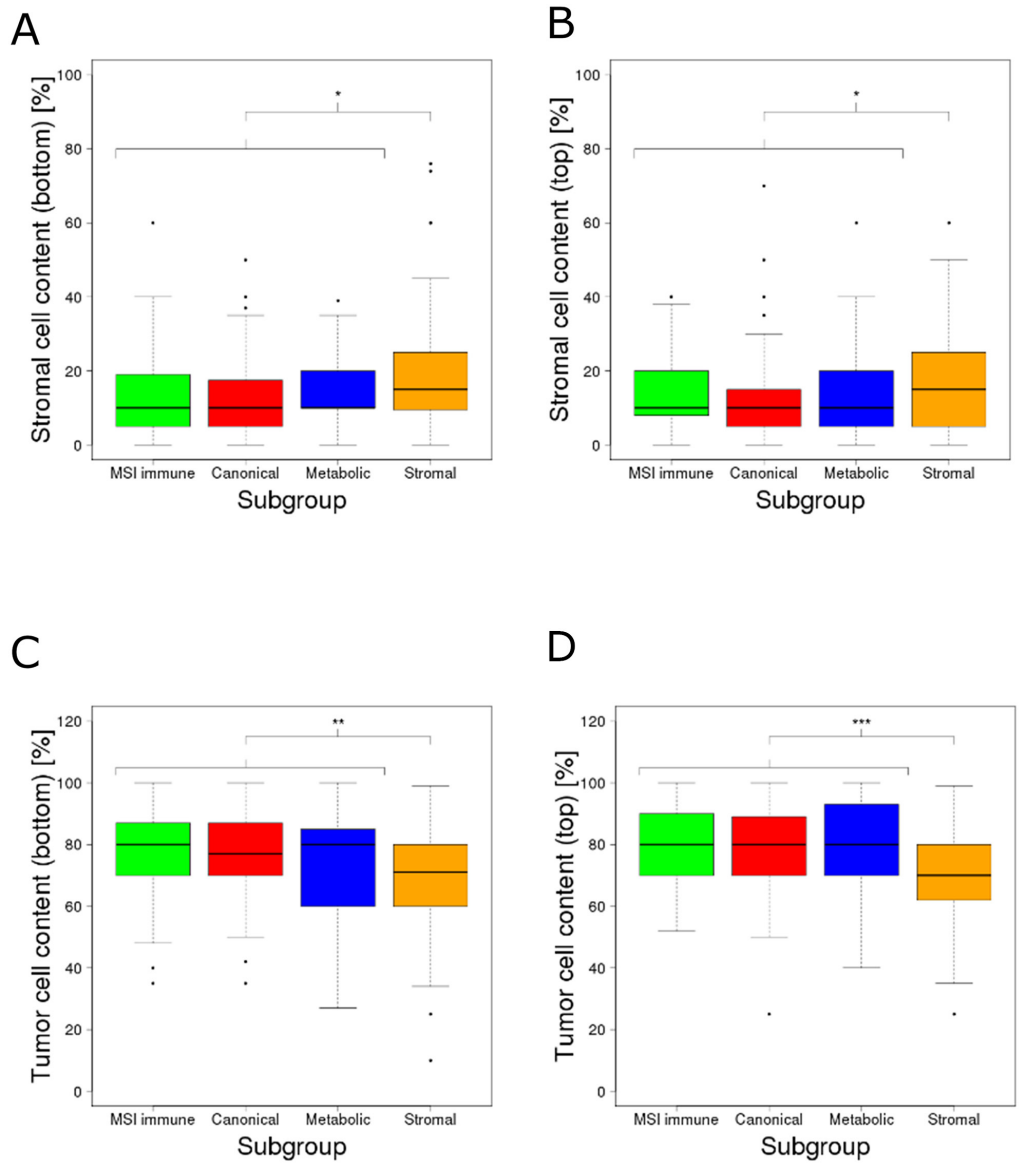
Estimated percentages of the content of stroma and tumor cells across the molecular subgroups Estimated percentages of stroma cell content at **(A)** the bottom of the tissue slide image and **(B)** the top of the tissue slide image; estimated percentages of tumor cell content at **(C)** the bottom of the tissue slide image and **(D)** the top of the tissue slide image. The content of stromal cells was significantly higher (bottom: p=0.013, top: p=0.031) whereas the content of tumor cells was significantly lower (bottom: p=0.0057, top: p=3.57e-5) in the stromal subgroup. The significance levels (^*^), (^**^) and (^***^) represent p-values between 0.05 and 0.01, between 0.01 and 0.001 and below 0.001 respectively.

Extracellular matrix (ECM) remodeling is a hallmark of tumor infiltration and metastasis [13, 14]. Compared to all other subgroups, stromal subgroup tumors were described by Guinney *et al.* as (i) more invasive, (ii) more metastatic, (iii) having reduced tumor cell content and (iv) being infiltrated by increased numbers of stromal cells (3). Isella *et al.* [15] and Calon *et al.* [16] also reported that a substantial fraction of transcriptomes from mesenchymal colorectal cancer subtypes could be explained by the stromal, rather than the epithelial tumor component. However, neither study mentioned the impact on ECM remodeling and its regulation by miRNAs. We investigated whether expression shifts in the miRNAs we identified and their corresponding ECM target genes (the combined 22 miRNAs and 91 target genes from the functional gene sets extracellular matrix organization and extracellular matrix disassembly) could be explained by stromal rather than tumor cells in samples of the stromal subgroup. We first assessed the differences of tumor and stromal cell proportions between samples of the stromal subgroup and the combination of samples of all other subgroups using image-based cell type estimates [2] (attribute details described in Supplementary 1.6). Stromal subgroup tumors contained 4.9% (bottom part of the image, p=0.013) and 3.4% (top part of the image, p=0.031) more stromal cells than the tumors from all other subgroups (Figure 3A–3B). In line, stromal subgroup samples contained 6.4% (bottom part of the image, p=0.0057) and 8% (top part of the image, p=3.57e-5) fewer tumor cells compared to samples from other subgroups (Figure 3C–3D). Stromal cells like fibroblasts, endothelial and immune cells can be adapted by the tumor to form a permissive tumor microenvironment supporting tumor protection, growth and invasion [17]. In response to cancer growth, quiescent fibroblasts residing in the stroma can be activated by growth factors such as TGF-β to become tumor-associated fibroblasts [17]. Tumor-associated fibroblasts are considered to be the main producers of ECM components, related proteins and growth factors in the tumor stroma [13, 17]. Similar to myofibroblasts in wounds or fibrotic tissues, tumor-associated fibroblasts are known to overexpress ACTA2, FAP and PDGFRB [18]. Indeed, ACTA2 (p=2.34e-32), FAP (p=1.32e-11) and PDGFRB (p=3.37e-21) expression were significantly up-regulated in the stromal subgroup tumors (Supplementary Figure 3). In summary, the proportion of stromal cells was higher in samples of the stromal tumor subgroup compared to samples of other subgroups. We found an up-regulation of known marker genes for tumor-associated fibroblasts, suggesting an increased contribution of these cells to the transcriptomic profile of stromal subgroup tumors.

### MicroRNAs regulate ECM target genes in the tumor stromal component

Our analysis identified 186 miRNA - target gene pairs containing 22 down-regulated miRNAs and 91 up-regulated, ECM-related target genes in the stromal tumor subgroup (Supplementary Table 9). In the following, the list of 22 miRNAs and the list of 91 target genes will be referred to as candidate miRNAs and candidate target genes, respectively. We were interested if the candidate target genes were rather expressed in the tumor cells or in the cells of the tumor microenvironment. For this, we investigated expression profiles of tumors and their microenvironment, separated into tumor cells and fibroblasts (“Calon” [19] and “Christensen” [20] datasets, only gene expression) or stromal cells (“Nishida” dataset [21], gene and miRNA expression; Scarpati dataset [22], only miRNA expression). Differential expression analysis identified 17 genes in the dataset of Christensen *et al.* [20], 51 genes in the dataset of Calon *et al.* [19] and 70 genes in the dataset of Nishida *et al.* [21] to be up-regulated in tumor-associated fibroblasts or stroma cells (Supplementary Table 10A-10C). Intersecting the 91 candidate target genes with the three lists of up-regulated genes in tumor-associated fibroblasts or stroma cells revealed significant enrichment in all three lists (p < 2e-5 in each list). We did not find any of the candidate target genes to be up-regulated in *tumor cells* in the investigated three datasets. To refine our list, we selected those candidate target genes which were up-regulated in at least one of the three datasets, leading to a refined list of candidates of ECM related target genes (n=76, “refined candidate target genes”). Among these refined candidates were genes encoding for integral ECM components (collagen, laminin and fibulin) as well as matrix degrading enzymes (particularly matrix metallopeptidases), matrix synthesis proteins (members of the lysyl oxidase family such as LOXL2) and genes involved in ECM-cell signaling (integrin ITGB1 and growth factor FGF2). To investigate the expression of the 22 candidate miRNAs in tumor and stromal cells, we analyzed their expression profiles in the datasets from Nishida and Scarpati. We confirmed that miR-192 and miR-17 were down-regulated in the stromal component compared to cancer cells, as reported by Nishida *et al.* [21] (Supplementary Table 11A) and that miR-17, miR-192 and miR-200c were down-regulated in stroma cells, as reported by Scarpati *et al.* [22] (Supplementary Table 11B). We performed gene set enrichment analysis on the 22 miRNAs among all down-regulated miRNAs in stromal cells in the Nishida dataset and observed a significant enrichment (p < 2e-5). These 22 candidate miRNAs were not significantly enriched among all down-regulated miRNAs in stromal cells in the Scarpati dataset (p = 0.18), however, a tendency towards enrichment was observed. None of the 22 candidate miRNAs were *up-regulated* in stromal cells in either dataset. As refined candidate miRNAs, we selected miR-192 and miR-17, which were significantly down-regulated in stromal cells in both datasets, and miR-200c, which was among the top 5 miRNA with the strongest down-regulation in stromal cells in the Scarpati dataset. Filtering the initial 186 miRNA - target gene pairs using the lists of refined candidate target genes and refined candidate miRNAs yielded 20 pairs with miRNAs that were down-regulated and target genes that were up-regulated in tumor-associated fibroblast or stromal cells respective. By integrating binding site predictions of miRNA – ECM target gene combinations that were not predicted by our combined model, we could specify 19 additional miRNA – target gene pairs and extended our result set to overall 39 candidate pairs. We identified SPARC, FGF2, DST, PLOD1, LOXL2, ITGB1, ITGAV, PXDN, FBN1, FN1, KDR, MMP2 and TGFB1 as potential target genes of miR-192; MMP2, FSCN1, LAMC1, TGFB1, DST, ETS1, FBN1, FGF2, FN1, ITGAV, ITGB1, PXDN and TIMP2 as feasible miR-17 targets; and ETS1, KDR, SERPINH1, TIMP2, NCAM1, FN1, FBLN5, DST, FGF2, ITGAV, ITGB1, PLOD1 and SPARC as potential miR-200c target genes (Table 1).

**Table 1:** Refined list of predicted candidate pairs of miRNAs and their target genes potentially involved in ECM remodeling

MiRNA	Gene symbol	Gene name	Identification method
hsa-mir-192	FGF2	Fibroblast Growth Factor 2	Combined Model
hsa-mir-192	ITGAV	integrin subunit alpha V	Combined Model
hsa-mir-192	LOXL2	lysyl oxidase-like 2	Combined Model
hsa-mir-192	ITGB1	integrin subunit beta 1	Combined Model
hsa-mir-192	PLOD1	procollagen-lysine, 2-oxoglutarate 5-dioxygenase 1	Combined Model
hsa-mir-192	PXDN	peroxidasin	Combined Model
hsa-mir-192	SPARC	Secreted Protein Acidic And Cysteine Rich	Combined Model
hsa-mir-192	DST	Dystonin	Combined Model
hsa-mir-192	FBN1	Fibrillin 1	Combined Model
hsa-mir-192	FN1	Fibronectin 1	Binding site analysis
hsa-mir-192	KDR	Kinase Insert Domain Receptor	Binding site analysis
hsa-mir-192	MMP2	Matrix Metallopeptidase 2	Binding site analysis
hsa-mir-192	TGFB1	Transforming Growth Factor Beta 1	Binding site analysis
hsa-mir-200c	ETS1	ETS Proto-Oncogene 1	Combined Model
hsa-mir-200c	KDR	Kinase Insert Domain Receptor	Combined Model
hsa-mir-200c	SERPINH1	Serpin Family H Member 1	Combined Model
hsa-mir-200c	TIMP2	TIMP Metallopeptidase Inhibitor 2	Combined Model
hsa-mir-200c	NCAM1	Neural Cell Adhesion Molecule 1	Combined Model
hsa-mir-200c	FN1	Fibronectin 1	Combined Model
hsa-mir-200c	FBLN5	Fibulin 5	Combined Model
hsa-mir-200c	DST	Dystonin	Binding site analysis
hsa-mir-200c	FGF2	Fibroblast Growth Factor 2	Binding site analysis
hsa-mir-200c	ITGAV	integrin subunit alpha V	Binding site analysis
hsa-mir-200c	ITGB1	integrin subunit beta 1	Binding site analysis
hsa-mir-200c	PLOD1	procollagen-lysine, 2-oxoglutarate 5-dioxygenase 1	Binding site analysis
hsa-mir-200c	SPARC	Secreted Protein Acidic And Cysteine Rich	Binding site analysis
hsa-mir-17	MMP2	Matrix Metallopeptidase 2	Combined Model
hsa-mir-17	FSCN1	Fascin Actin-Bundling Protein 1	Combined Model
hsa-mir-17	LAMC1	Laminin Subunit Gamma 1	Combined Model
hsa-mir-17	TGFB1	Transforming Growth Factor Beta 1	Combined Model
hsa-mir-17	DST	Dystonin	Binding site analysis
hsa-mir-17	ETS1	ETS Proto-Oncogene 1	Binding site analysis
hsa-mir-17	FBN1	Fibrillin 1	Binding site analysis
hsa-mir-17	FGF2	Fibroblast Growth Factor 2	Binding site analysis
hsa-mir-17	FN1	Fibronectin 1	Binding site analysis
hsa-mir-17	ITGAV	integrin subunit alpha V	Binding site analysis
hsa-mir-17	ITGB1	integrin subunit beta 1	Binding site analysis
hsa-mir-17	PXDN	peroxidasin	Binding site analysis
hsa-mir-17	TIMP2	TIMP Metallopeptidase Inhibitor 2	Binding site analysis

Taken together, our re-analysis of 4 datasets separating expression from the stromal and tumor cell components of colorectal cancers have paired over-expressed target genes responsible for ECM (dis)assembly and regulation with the down-regulation of miR-192, miR-17 and miR-200c in tumor-associated fibroblasts. This gene regulatory network may be pivotally involved in ECM remodeling in the stromal colorectal tumor subgroup and might be highly relevant for colorectal cancer cell invasion and metastasis.

### Enforced expression of miR-192, miR-17 and miR-200c in fibroblasts suppresses predicted target gene expression *in vitro*

**Figure 4:**
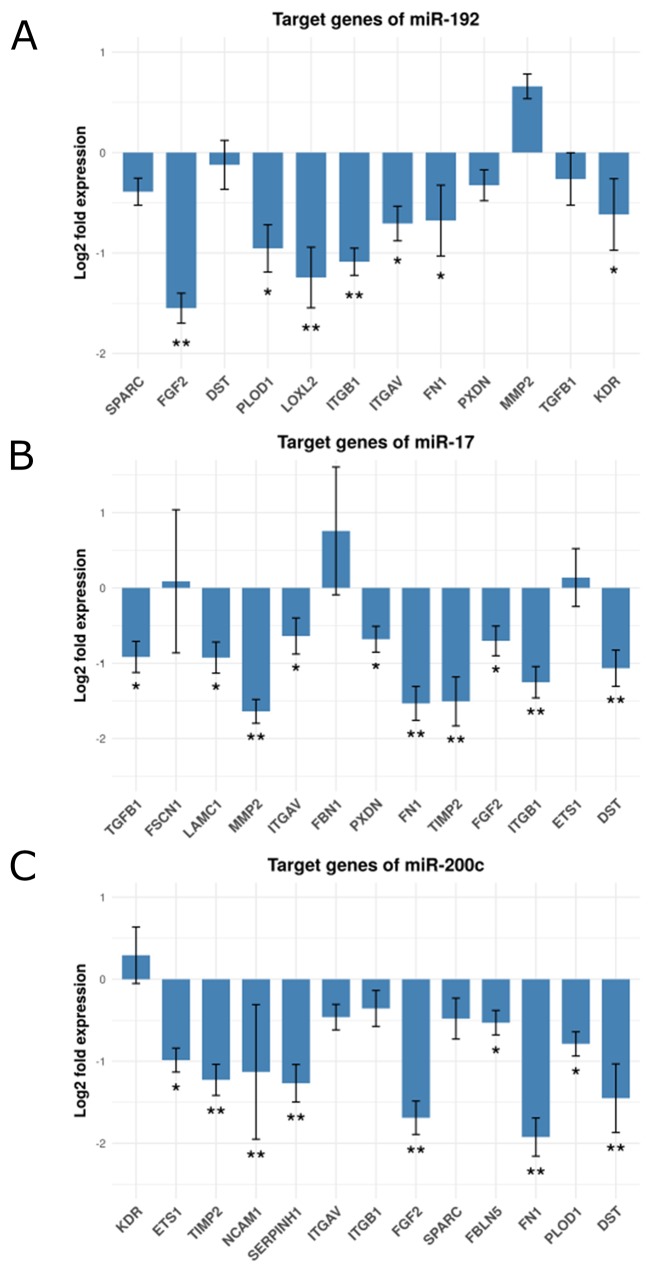
Fold change expression of the candidate genes after miRNA transfection in fibroblasts Shown are bar-plots of (log2 transformed) fold expression values (miRNA transfected versus miRNA controls), **(A)** Target genes of mir-192, **(B)** Target genes of mir-17 and **(C)** Target genes of mir-200c. (^*^) and (^**^) represent log2 fold changes between -0.5 and -1 and between -1 and -2 respective. All experiments were performed in three (n=3) technical replicates.

We set out to experimentally validate our computationally derived hypothesis involving the miRNAs and ECM-remodeling target genes identified. We tested the suppressive effect of miR-192, miR-17 and miR-200c on their respective target genes by transfecting miRNA mimics or a control mimic into human fibroblasts and subsequently assessed target gene expression using qPCR. ITGAV, ITGB1, LOXL2, PLOD1, FGF2, FN1 and KDR were all down-regulated by enforced expression of miR-192. We observed a negative, yet not significant effect on the expression of SPARC, DST, PXDN and TGFB1. Surprisingly, miR-192 had a positive effect on the expression of MMP2 (Figure 4A and Supplementary Table 12A). The expression of FBN1 was below the detection level. TGFB1, LAMC1, MMP2, ITGAV, PXDN, FN1, TIMP2, FGF2, ITGB1 and DST were down-regulated after enforced expression of miR-17. We observed a positive effect on FBN1 and only very little effect on FSCN1 and ETS1 expression (Figure 4B and Supplementary Table 12B). Enforcing miR-200c expression caused down-regulation of FN1, FBLN5, ETS1, TIMP2, SERPINH1, FGF2, PLOD1, NCAM1 and DST. The expression of ITGAV, ITGB1 and SPARC were also down-modulated by miR-200c, but were below the threshold. KDR showed a slightly positive alteration (Figure 4C and Supplementary Table 12C). In summary, we could validate 67% of our computationally predicted miRNA - target gene pairs by cell culture experiments. 85% of our 20 ECM candidate genes are targeted by at least one of the three candidate miRNAs. Remarkably, FGF2 and FN1 are down-regulated by all three miRNAs. We present 17 miRNA-regulated genes expressed in the stromal component of colorectal cancers and involved in ECM remodeling as potentially important in tumorigenesis.

### MiRNAs miR-192, miR-17 and miR-200c regulate ECM target genes on the protein level

We investigated the effect of miR-192, miR-17 and miR-200c on the protein expression of their individual targets to elucidate if the miRNAs regulate ECM remodeling also on the protein level. We tested 28 miRNA – target gene pairs where the data was available for inverse correlation of miRNA expression and protein abundance (see Supplementary 1.7 for details). We observed significant negative correlation for 10 pairs: miR-200c and FN1 (p=2.94e-4), miR-17 and ITGAV (p=5.08e-4), miR-17 and FBN1 (p=1.26e-3) miR-17 and FSCN1 (p=3.74e-3), miR-17 and ITGB1 (p=4.17e-3), miR-17 and LAMC1 (p=7.86e-3), miR-200c and ITGAV (p=9.03e-3), miR-200c and ITGB1 (p=1.26e-2), miR-17 and MMP2 (p=2.6e-2), and miR-192 and SPARC (p=4.93e-2). A tendency of negative correlation was observed for miR-200c and SERPINH1 (p=5.49e-2), miR-192 and FN1 (p=5.56e-2) and miR-192 and MMP2 (p=5.86e-2) (Supplementary Table 13). Afterwards, we compared the correlation coefficients of the 28 miRNA – target gene pairs with the correlation coefficients of all possible combinations of all other non-candidate miRNAs and proteins (n=2,987,138) and observed a significant lower correlation of our candidate pairs (p=3.68e-6, Student’s *t*-test). These results demonstrate that miR-192, miR-200c and miR-17 are not only inversely correlated to their ECM target genes but also to their expressed proteins.

As MMP2 is known to be a substantial player in ECM remodeling and associated with tumorigenesis [23], we measured the protein level of MMP2 in the supernatant of miRNA - transfected MRC5 and CCD-18Co fibroblasts using western blots to confirm our computational findings. We observed a reduced relative abundance of MMP2 in both cell lines after transfection with miR-200c, miR-17 or miR-192 compared to a control transfection (see Supplementary Figure 4). The results in both cell lines indicate that the protein abundance of MMP2 is negatively affected by the identified miRNAs.

### Enforced miR-192, miR-17 and/or miR-200c expression in colon fibroblasts reduces invasive capacity of co-cultured colorectal cancer cells

**Figure 5:**
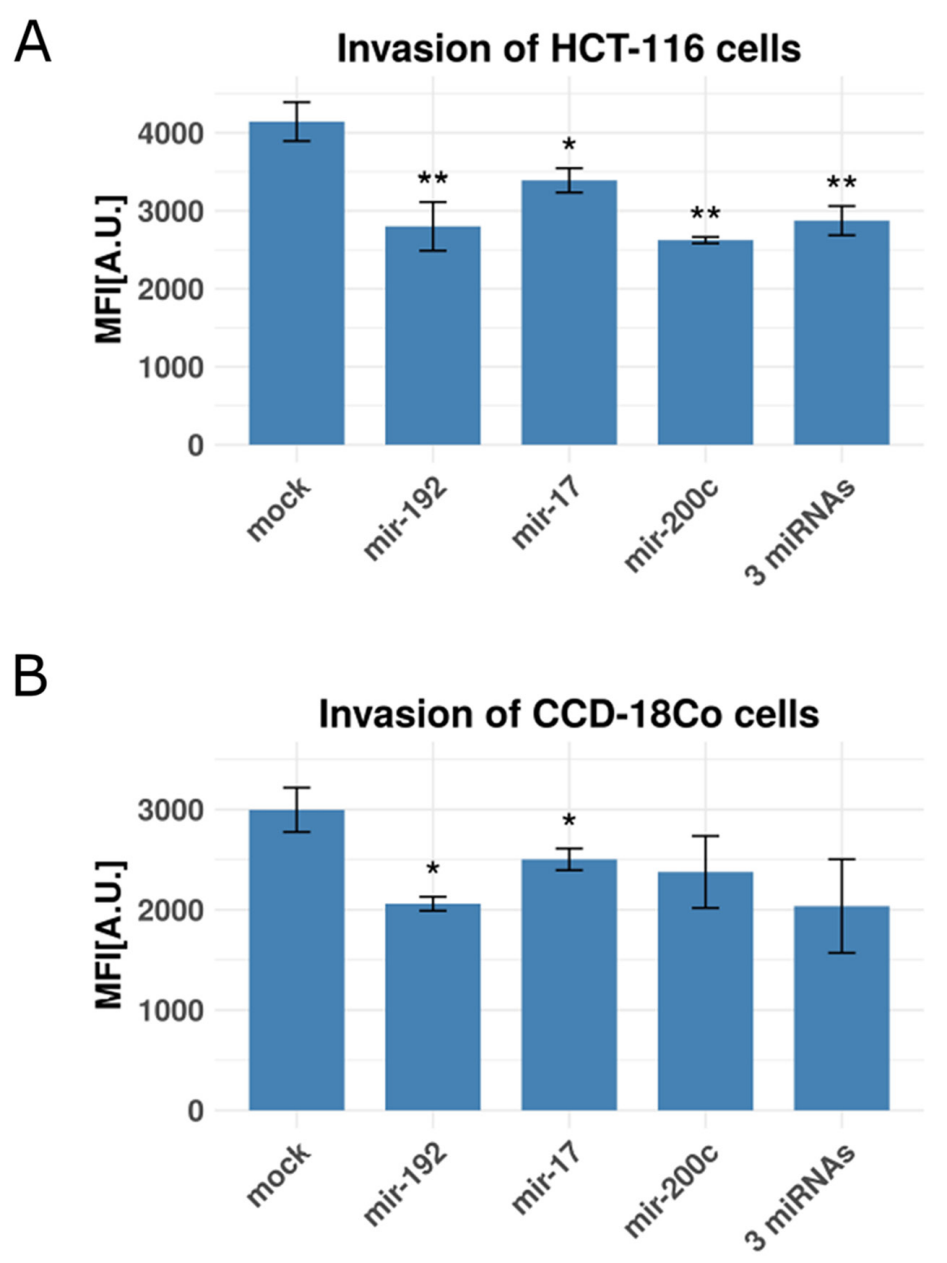
Invasion rates of colon cancer cells and fibroblasts with and without miRNA transfection Shown are bar plots of invasion rates as relative mean fluorescence intensities (MFI) in arbitrary units [A.U.]. **(A)** Co-cultured HCT-116 cancer cells and CCD-18Co fibroblasts (with CCD-18Co cells in the lower chamber) transfected with mir-192, mir-17, mir-200c and a combination of all three miRNAs using a mock-transfection as control. **(B)** CCD-18Co fibroblasts, transfected with mir-192, mir-17, mir-200c and a combination of all three miRNAs using a mock-transfection as control. (^*^), (^**^) and (^***^) represent p-values between 0.05 and 0.01, between 0.01 and 0.001 and below 0.001 respective. All experiments were performed in three (n=3) technical replicates.

The question remains whether the miRNA- mediated down-regulation of the 17 target genes in tumor-associated fibroblasts could affect the invasive capacity of the adjacent cancer cells. To experimentally test our hypothesis, we set up a Boyden-chamber assay with HCT-116 colon cancer cells in the inner chambers and CCD-18Co colon fibroblasts in the outer chambers and measured cancer cell invasion as mean fluorescent intensity. Fibroblasts were selectively transfected with mimics of miR-192, miR-17, miR-200c, a combination of all three mimics or a mock control mimic. HCT-116 cell invasion was significantly reduced by co-culture with colon fibroblasts expressing miR-200c (p=7.5e-3), miR-192 (p=4.99e-3), miR-17 (1.66e-2) or all 3 miRNA (p=2.7e-3, Figure 5A). Enforced expression of either miR-192 (p=0.01) or miR-17 (p=0.04) also significantly reduced the invasive activity of CCD-18Co fibroblasts. This observation suggests that ECM remodeling initiated by miRNA regulation may affect both stromal and epithelial colorectal tumor components *in vivo*. Enforced expression of miR-200c alone (p=0.07) or the combination of all 3 miRNAs (p=0.052) did not significantly impact the invasive activity of CCD-18Co fibroblasts, although we observed the same tendency towards reduced invasion (Figure 5B). To exclude any side effects of proliferation on the invasion of co-cultured cancer cells, we performed XTT proliferation assays. HCT-116 cells co-cultured with the supernatant of CCD-18Co fibroblasts that were transfected with miR-17, miR-200c or miR-192 beforehand showed no significant alteration of proliferation compared to a control transfection (Supplementary Figure 5). Further investigations using sophisticated 3D or *in vivo* tumor models are needed to show if the concept of migratory track preparation by tumor-associated fibroblasts proves relevant also for colorectal cancers, but are beyond the scope of this study. Our experimental data show that miR-192, miR-17 and miR-200c expression in fibroblasts impacts the migratory capacity of co-cultured colon tumor cells, strengthening the computationally predicted functional association with the increased metastatic quality of cancers of the stromal subgroup.

## DISCUSSION

We established a piecewise linear model to observe non-linear regulation of gene expression by miRNAs. The method is implemented as an R (https://www.r-project.org/) package named MiRNA-RIP and is freely available at http://www.leibniz-hki.de/en/mirnarip.html. By including molecular subgroups of colorectal cancer established by Guinney *et al.* [3] in our analysis, we elaborated subgroup-specific miRNAs, their corresponding target genes and regulated functional gene sets. We identified miR-192, miR-17 and miR-200c as regulators of genes involved in extracellular matrix remodeling in the stromal subgroup of colon cancer.

### MiRNAs regulate ECM remodeling in the tumor microenvironment

**Figure 6:**
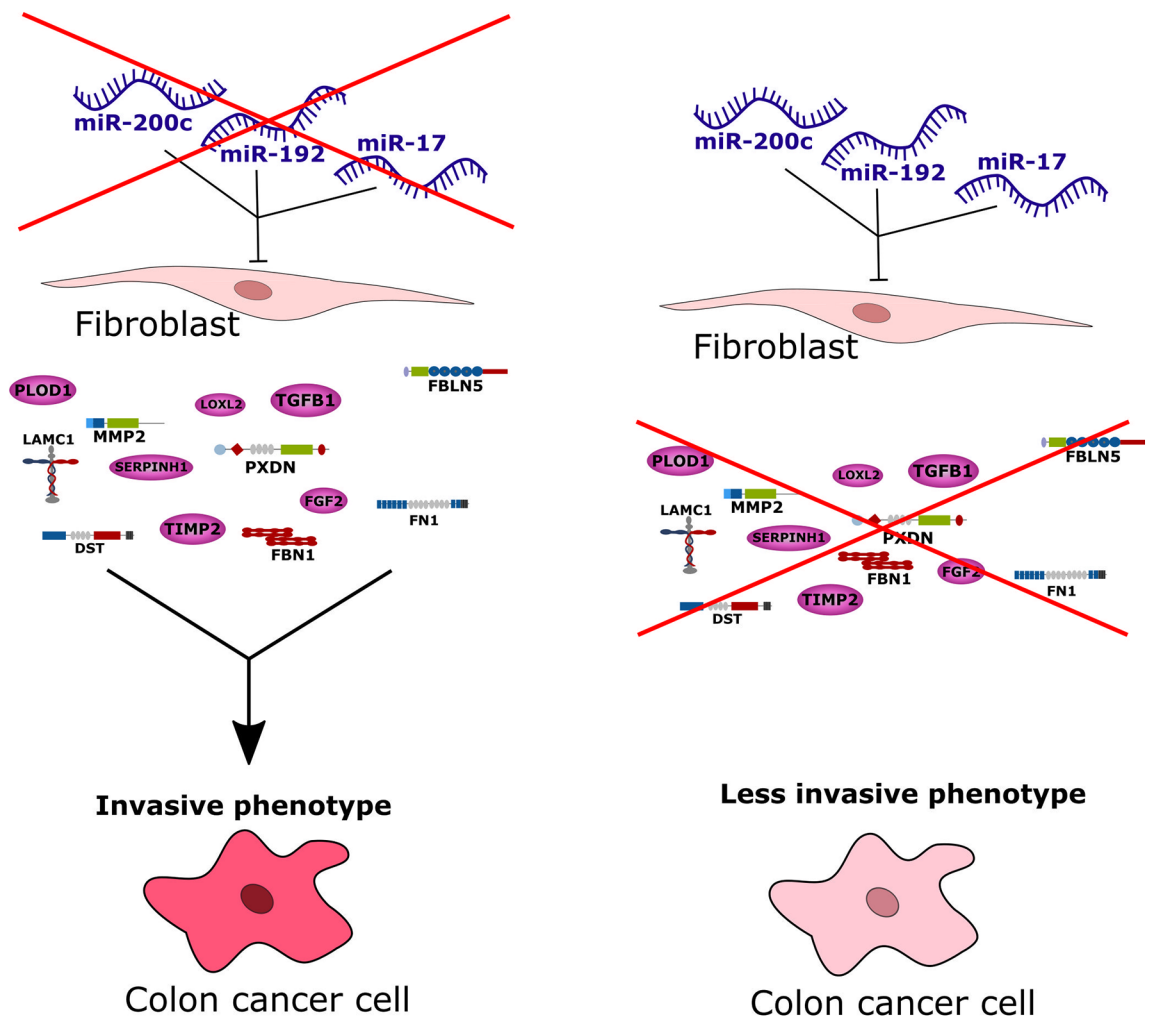
Conceptional summary of the study The stromal subgroup of colorectal cancer exhibits high metastatic potential of cancer cells and thus this subgroup has the worst survival prognosis compared to all other subgroups. Based on our study we conclude that by down-regulation of miR-200c, miR-192 and miR-17, tumor-associated fibroblasts can produce more ECM-related and invasion-promoting factors leading to an increased invasion of cancer cells and thus a highly invasive phenotype. Admission of these three miRNAs to tumor-associated fibroblasts in turn blocks transcription or translation of invasion-promoting proteins leading to a less invasive phenotype.

Most research on miRNAs in cancer to date focus on their specific roles in the cancer cells. A literature analysis revealed several colorectal cancer studies investigating the transcriptional discrepancy between cancer cells and their surrounding stromal cells [15–17]. We confirmed from publicly available imaging data that the molecularly defined stromal colorectal cancer subgroup had significantly increased proportions of infiltrated stromal cells. Hence, it was quite likely that the infiltrating stromal cells, or particularly tumor-associated fibroblasts, could explain the distinct gene expression pattern in this subgroup. We investigated data from several published miRNA and mRNA transcriptome studies of sorted stromal and cancer cells from colon tumors [19–22], and validated the hypothesis that up-regulated genes associated with ECM remodeling are more strongly expressed in the stromal rather than epithelial cancer cell component of tumors. In line with this, we identified their putative miRNA regulators to be significantly down-regulated in stromal cells. We experimentally validated these computational findings and observed down-regulation of 85% of the predicted ECM remodeling target genes in fibroblasts after enforced expression of miR-192, miR-17 or miR-200c. Enforced expression of these miRNA regulators in fibroblasts also repressed invasive activity of co-cultured colorectal cancer cells. We argue that the three identified miRNAs down-regulate target genes associated with ECM remodeling in fibroblasts, and the down-regulation of these miRNA regulators in stromal subgroup colorectal tumors could account for the heightened invasive or metastatic capacity of their cancer cells, sketched in Figure 6. Interestingly, we also observed a reduction of fibroblast migration after enforced expression of miR-192 or miR-17. This is in line with observations by Gaggioli *et al.* that squamous cell carcinoma cells migrate within tracks through the extracellular matrix behind tumor-associated fibroblasts [24] and similarly by Li *et al.* that adenoid cystic carcinoma cells follow the track behind tumor-associated fibroblasts [25]. This aspect could be another, indirect contribution of tumor-associated fibroblasts to tumor cell migration.

### Tumor-suppressive miR-192, miR-17 and miR-200c are known regulators in cancer

Some evidence that miR-192, miR-17 and miR-200c play a role in cancer pathophysiology has been described in the literature. Geng *et al.* [26] reported that miR-192 expression progressively decreased with increasing colorectal cancer stage, being lowest in stage IV tumors, and demonstrated in mouse models that enforced expression of miR-192 inhibits metastatic colonization of the liver. In medulloblastoma, miR-192 has been described as a suppressor of metastasis and inhibitor of cell adhesion to ECM components via integrins such as ITGB1 and ITGAV [27]. MiR-200c is a regulator of epithelial-to-mesenchymal transition which targets the transcriptional repressors of E-cadherin, ZEB1 and ZEB2 [28], suggesting a tumor suppressive function. The miR-200c - ZEB1 axis was also linked to invasive activity in breast cancer cells [29]. Hur *et al.* [30] compared miR-200c expression in primary colorectal tumors, and reported that lower miR-200c expression correlated with increased metastatic activity. They used *in situ* hybridization to show that miR-200c expression gradually decreased from the lumen to the submucosa in primary colorectal tumors, contributing to a higher metastatic potential at the invasive tumor front [30]. Chen *et al.* [31] associated elevated miR-200c expression in colon cancer cells with lymph node metastasis, invasion and resistance to cytarabine treatment. The genomic locus of the miR-17∼92 cluster is a direct target of the MYC family oncogenes. The locus has been shown to be amplified in tumor cells and its overexpression has been associated with tumor-promoting processes in several malignancies (reviewed in [32]). Despite its known tumor-promoting abilities, the expression of the miR-17∼92 cluster in prostate cancer cells has been shown to inhibit cell migration and epithelial-to-mesenchymal transition, suggesting a tumor suppressive role in some cases [33]. Expression of miR-17 in colorectal tumors has been reported to increase with cancer severity, from adenoma to adenocarcinoma [34]. Strikingly, expression was confined to the epithelial cancer cell component of these tumors. These studies do not contradict our observations, since we detect the miRNA down-regulation specifically in the tumor-associated stromal cells, and, in relation to the stroma, expression in the epithelial tumor cells. As a future aspect, it would be intriguing to investigate the expression of miR-17 and miR-200c in the surrounding stroma of the reported tumor types, and in particular for tumors with amplification or overexpression of the MYC oncogene family.

### ECM remodeling genes contribute actively to tumor invasion

The identified target genes have been reported in the literature in the context of invasion and metastasis. LOXL2 is a member of the lysyl oxidase gene family which catalyzes the crosslinking of extracellular collagen and elastin resulting in increased ECM stiffness and subsequent activation of the kinases FAK and SRC, which enable tumor cells to proliferate and invade. Furthermore, LOXL2 is involved in metastasis [35–37]. LOXL2 expression is increased in a number of tumor types, including colorectal cancer [38] where it is also a marker for poor prognosis [37]. Specific knockdown experiments demonstrated a stimulating role of LOXL2 during cancer progression. Conditioned medium from fibroblasts, which were extracted from the gastric wall of diffuse-type gastric carcinoma patients and transfected with LOXL2 siRNA, was co-cultured with gastric cancer cells. While untreated conditioned medium increased the migration and invasion, LOXL2 siRNA treatment of cancer-associated fibroblasts significantly decreased the migration and invasion of gastric cancer cells, underlining the tumor promoting role of LOXL2 in tumor-associated stroma cells [39]. Silencing of LOXL2 in cancer cells led also to a decrease of invasiveness in pancreatic [40] and gastric cancer [41] *in vitro* models.

The genes ITGB1 and ITGAV are members of the integrin gene family. Their gene products can form a heterodimeric integral membrane protein, which serves as a receptor for vitronectin, fibrinogen and other ligands. While overexpression of ITGAV was linked to progression and perineural invasion in colorectal cancer [42], ITGB1 expression in tumors was associated with shortened overall survival and shortened disease-free survival in a large cohort of patients with colorectal tumor [43]. RNAi-mediated knockdown of ITGB1 in human colorectal cancer cells significantly reduced the proliferation and invasion and resulted in slower tumor growth rates and smaller tumor volumes compared to control transfections in xenograft mouse models [44]. In MCF-7 breast cancer cells, knocking down ITGAV led to a significant inhibition of matrigel invasion [45].

Tissue Inhibitor of Metalloproteinases 2 (TIMP2) can bind various matrix metalloproteinases (MMPs), a large family of ECM-degrading endopeptidases. TIMP2 inactivates them irreversibly to prevent excessive destruction of the ECM network and to maintain tissue homeostasis. Roca *et al.* [46] performed an immunohistochemical analysis of TIMP2 in colorectal cancer samples and showed a specific staining not only in epithelial tumor cells but also in the ECM of the stromal compartment. High expression of TIMP2 was associated with bad outcome in colorectal cancer [46] and shortened disease-free and overall survival in human breast cancer [47]. SiRNA-mediated knockdown of TIMP2 in HCT-116 colon cancer resulted in a reduction of invasion of HCT-116 cells *in vitro* [48].

The large family of matrix metallopeptidases (MMPs) plays a key role in tumor progression and metastasis as they actively drive proteolysis of physical barriers like the basement membrane which allows tumor cells to invade the surrounding tissue. They are also involved in epithelial-to-mesenchymal transition by shedding E-cadherin and cleavage of ECM-bound growth factors and cytokines (reviewed in [49]). MMP2 specifically degrades collagen IV, a major component of the basement membrane, but also elastin, fibronectin and laminin. Its enzymatic activity is mainly regulated by a major MMP antagonist, TIMP2 which prevents MMP2 from excessively ECM degrading. In the context of colorectal cancer, the mean activity of MMP2 was found to be around ten times higher in tumor tissue compared to normal mucosa [50]. Interestingly, Wiese *et al.* demonstrated that MMP2 expression was rather linked to stromal than epithelial tumor cells [51]. Dong and co-workers showed that shRNA-mediated knockdown of MMP2 in HT-29 and SW480 colon tumor cells clearly reduced their invasion capabilities in Boyden-chamber assays [52]. Further knockdown experiments using colorectal cancer cells revealed invasion or migration promoting roles for SPARC [53], FN1 [54] and FSCN1 [55]. The biological relevance of other miRNA target genes is discussed in Supplementary 2.3.

In summary, the literature evidenced the identified miRNA-regulated ECM genes to have an invasion promoting function in the tumor microenvironment of colorectal cancer and other tumor entities.

## MATERIALS AND METHODS

### Acquisition of miRNA target genes

As a resource for miRNA target genes, we downloaded experimentally validated, manually curated miRNA - target gene interactions from DIANA-TarBase v7.0 [11] and selected miRNA - target gene pairs from experiments performed in cells of human origin, irrespective of the applied detection method. After filtering, the dataset comprised of 322,145 unique miRNA - target gene pairs. Mature miRNA identifiers (typically with -3p or -5p specification) used in TarBase were mapped to pre-miRNA identifiers used in the experimental data according to the miRNA annotation data retrieved from miRBase [56]. Target genes of mature miRNAs belonging to the same pre-miRNA identifier were merged resulting in a unique set of target genes per pre-miRNA.

### The TCGA colon adenocarcinoma dataset and colorectal cancer subgroup definitions

The TCGA colon adenocarcinoma dataset [2] used to establish our method comprises 271 tumor and eight control samples, for which both mRNA and miRNA expression data were available. Tumor samples originated from 146 male and 117 female patients with a median age at diagnosis of 67 years. Pathological TNM tumor staging based on the TNM-system [57] classified 42 tumors as stage I, 104 tumors as stage II, 72 tumors as stage III and 37 tumors as stage IV. Median overall survival was 409 days with a death rate of 21.2%. Recurrence-free survival averaged 401 days with a recurrence rate of 18.5%. The maximal follow-up period was 11.7 years. For our analysis, we used the molecular subgroup definitions identified by Guinney *et al.* [3], who combined different classifying methods and multiple colorectal cancer datasets (including the TCGA dataset) to define the subgroups, microsatellite instability immune (MSI immune), canonical, metabolic and mesenchymal, which have distinct clinical courses, expression profiles and pathway enrichments.

The MSI immune subgroup is characterized by microsatellite instability, immune cell infiltration and activation of immune response gene sets. Approximately 37% of the samples investigated by Guinney *et al.* [3] were assigned to the canonical subgroup, which was characterized by increased WNT and MYC signaling activity. Patients of this subgroup had the best overall survival. The miR-17–92 cluster (a direct MYC target, which includes miR-17, miR-18a, miR-19a, miR-20a, miR-19b-1, miR-92a-1), was overexpressed in canonical subgroup tumors. Biochemical pathways, such as glucose and amino acid metabolism, were dysregulated in the metabolic tumor subgroup, which also harbored a high proportion of KRAS and APC mutations and expressed low levels of miR-143 and six members of the let-7 family (let-7c, let-7e, miR-100, miR-125b-1, miR-125b-2 and miR-99a), which were associated with elevated KRAS expression. The metabolic subgroup was also epithelial in nature. Tumors from patients with the worst overall and relapse-free survival belonged to the mesenchymal subgroup, which also contained the highest proportion of stage III and IV tumors. MiRNAs previously shown to be tumor suppressive (miR-148a, miR-192 and members of the miR-200 family) were down-regulated in mesenchymal tumors. Guinney *et al.* [3] associated miR-192 and miR-200 family down-regulation with epithelial-to-mesenchymal transition and miR-148a down-regulation with TGF-β signaling and matrix remodeling, all processes enriched in the mesenchymal tumor subgroup. TCGA samples with distinct molecular subgroup assignments defined by Guinney *et al.* [3] included 40 (MSI immune), 75 (canonical), 34 (metabolic), and 66 (mesenchymal) samples. Because our data show the mesenchymal tumor subgroup to be enriched with tumor-associated stromal cells, we use the term “stromal subgroup” in the text.

### The TCGA prostate adenocarcinoma dataset

For performance testing and model comparison, we applied our method to another TCGA dataset comprising of 404 prostate adenocarcinoma samples for which miRNA and gene expression data were available [58]. The datasets were downloaded from the UCSC Cancer Browser (https://genome-cancer.ucsc.edu/proj/site/hgHeatmap). We used RNA sequencing-based gene expression Level 3 data which were generated from sequences of the Illumina HiSeq 2000 RNA sequencing platform and processed using the RNASeq version 2 pipeline (https://wiki.nci.nih.gov/display/tcga). MiRNA expression was measured using the Illumina HiSeq 2000 and the Illumina Genome Analyzer platform. The data were provided as mapped reads per million miRNA (RPMM). Supplementary Table 1 provides an overview of the used datasets.

### Expression data of miRNA transfection experiments for validating the model predictions

To validate potential miRNA target genes predicted by our model, published gene expression data of miRNA transfection experiments were downloaded from the Gene Expression Omnibus (https://www.ncbi.nlm.nih.gov/geo). MiRNA mimics or antagomiRs were transfected into various human cancer cell lines. Depending on the individual experimental design, different reagent concentrations and optional treatment of the cells prior to transfection were applied. Gene expression was measured at different time points using microarrays or RNA sequencing. We used data from 41 individual experiments of 12 different miRNAs: miR-17 (Ivanovska *et al.* [59], Doebele *et al.* [60], Martin *et al.* [61], Linsley *et al.* [62]), miR-20 (Ivanovska *et al.* [59], Linsley *et al.* [62]), miR-106a (Ivanovska *et al.* [59]), miR-106b (Ivanovska *et al.* [59], Linsley *et al.* [62]), miR-92a (Borkowski *et al.* [63]), miR-192 (Linsley *et al.* [62], Georges *et al.* [64]), miR-215 (Linsley *et al.* [62], Georges *et al.* [64]), miR-16 (Linsley *et al.* [62]), miR-26b (Chen *et al.* [65]), miR-145 (Gregersen *et al.* [66]), miR-1 (Suzuki *et al.* [67]), and miR-7 (Hausser *et al.* [68]). Additionally, we collected data from 36 experiments of 19 different miRNAs to confirm the results of the analysis of the TCGA prostate adenocarcinoma dataset: miR-205 (Gandellini *et al.* [69], Boll *et al.* [70]), miR-29b (Takayama *et al.* [71]), miR-135a (Kroiss *et al.* [72]), miR-145 (Ozen *et al.* [73], Kinoshita *et al.* [74], Nohata *et al.* [75]), miR-221 (Kneitz *et al.* [76]), miR-224 (Kristensen *et al.* [77]), miR-452 (Kristensen *et al.* [77]), miR-23b (Kinoshita *et al.* [74]), miR-24 (Kinoshita *et al.* [74]), miR-27b (Kinoshita *et al.* [74], Hudson *et al.* [78]), miR-143 (Kinoshita *et al.* [74]), miR-222 (Fuse *et al.* [79]), miR-31 (Fuse *et al.* [78], Lin *et al.* [80]), miR-106b (Hudson *et al.* [81]), miR-1 (*Hudson et al.* [78]), miR-206 (Hudson *et al.* [78]), miR-99a (Sun *et al.* [82]), miR-135b (Aakula *et al.* [83]), and miR-130a (Boll *et al.* [70]). Details about used cell lines, conditions, time points, miRNAs and experimental designs are listed in Supplementary Table 2.

### Cancer and stroma cell expression datasets

To investigate gene and miRNA expression in tumor and tumor associated stroma cells, we investigated several publicly available datasets. Calon and colleagues [19] collected tissues containing both cancer and non-cancer cells from six colorectal tumors. The tissue samples were minced and single cells were collected by sequential filtering. The filtered cells were stained with FAP- and hEPCAM antibody followed by fluorescence activated cell sorting (FACS) to obtain fibroblasts of the stroma and epithelial tumor cells. Cell type specific gene expression profiles were obtained using microarrays (Affymetrix HT HG-U133+). Furthermore, we analyzed expression data from the study of Nishida and co-workers [21]. Nishida et al. collected stromal and epithelial RNA samples from 13 colorectal cancers and four control tissues using laser capture microdissection for cell type separation. Both miRNA and gene expression were profiled using microarrays (gene expression: Agilent-014850, miRNA expression: Agilent-019118) for both cell types. Christensen *et al.* [20] also employed laser capture microdissection on colorectal tumor samples and compared the gene expression data from laser-dissected tumor cells and cultured tumor-associated fibroblasts. Finally, the dataset published by Scarpati and colleagues [22] provided miRNA expression profiles for tumor and stroma tissues which were microdissected from 57 surgical specimens taken from colorectal cancer patients. Supplementary Table 3 provides details about the datasets from these cell type specific experiments.

### Statistical analysis and model implementation

Statistical analysis, data processing and setting up the optimization problem were all performed using R (https://www.r-project.org/, version 3.2.4). The Gurobi Optimizer (http://www.gurobi.com, version 6.5.2, academic license) was used to solve both the linear and the piecewise linear model.

### Data pre-processing

We removed miRNAs and genes having no measurable expression intensities (RPKM=0 for all samples). Among the collection of miRNA target genes from TarBase, we selected only interactions measured in cells of human origin and mapped miRNA identifiers from TarBase to miRNA identifiers used in the TCGA dataset. For each miRNA - target gene pair, Pearson’s correlation of miRNA- and target gene expression was computed, and only pairs with a negative correlation were considered for further analysis. Finally, both miRNA expression and gene expression datasets were z-normalized.

### Cell type-specific expression analysis of extracellular matrix-related target genes

To support the hypothesis that extracellular matrix (ECM)-related genes identified in our analysis are particularly expressed by tumor-associated fibroblasts or stroma cells and not by tumor cells, we investigated expression data from four different studies [19–22], which we refer to by the paper first author for clarity. The Calon dataset provided gene expression profiles from fibroblasts and tumor cells separated by fluorescence-activated cell sorting (FACS). Microdissection was used so separate tumor and stromal cells for gene (Christensen, Nishida) and miRNA (Nishida, Scarpati) expression profiles. Tumor-associated fibroblasts were separated from primary intestinal colon carcinoma samples using cell culture protocols in the study from Christensen. We tested genes and miRNAs for differential expression in tumor and non-tumor cells in each dataset individually. Pairwise Student’s *t*-test followed by multiple-testing correction using the Benjamini-Hochberg method was performed to test for differential expression between the specific cell types. Gene-set enrichment analysis was performed using the “runGSA”-method from the piano package [84]. We chose the parameters “mean” as gene set statistics, “fdr” as multiple-testing correction method and repeated data randomization (parameter: “nPerm”) 50,000 times. Only gene sets with adjusted p-values of at most 0.05 for distinct directionality (“pAdjDistinctDirDn” and “pAdjDistinctDirUp”) were considered to be significantly enriched. See Figure 1B for an illustrated workflow.

### Identification of additional miRNA target genes with binding site predictions

For each candidate miRNA, we determined additional potential targets from the pool of 20 ECM target genes which resulted from the cell type specific expression analysis. We searched for miRNA binding-sites with the prediction tools MirWalk [85], PicTar [86], PITA [87], RNA22 [88] and TargetScan [89] (conserved and non-Conserved). We considered a binding site predicted with at least one prediction tool as criterion for inclusion of the candidate gene as additional target gene of the respective miRNA. An overview over the additional target genes and identified binding sites with the corresponding prediction tool is presented in Supplementary Table 4.

### Cell lines and cell culture

The human fibroblast cell lines CCD-18Co and MRC5 were purchased from ATCC (ATCC, VA, USA). CCD-18Co cells were cultured in DMEM medium with high glucose (1 g/L) and sodium pyruvate (110 mg/L) (Gibco, Carlsbad, CA, USA) supplemented with 10% FCS superior (Biochrom, Berlin, Germany) without antibiotics at 37°C and 5% CO2. The cells were genotyped and tested for mycoplasma contamination. MRC5 cells were cultured in MEM medium (Gibco) supplemented with 10% FCS superior without antibiotics at 37°C and 5% CO2. HCT-116 cells were cultured in McCoy’s 5a Medium Modified (Gibco) + 10% FCS superior without antibiotics at 37°C and 5% CO2. Cells were tested for mycoplasma contamination.

### MiRNA transfection

Fibroblasts were seeded in 6 well plates and cultured until they reached 80% confluence. Transfection was performed using Lipofectamine RNAiMAX reagent (Life technologies, CA, USA), which resulted in high transfection efficacy of 80% and low effect on cell viability for CCD-18Co cells (Supplementary Figure 6). The same protocol was used for MRC5 cells. Cells were transfected with 50 nM mimic control-1 (ath-miR416), miR-192, miR-17 or miR-200c, respectively. Two days post transfection cells were harvested and cell pellets were shock-frozen and stored at -80°C until further usage.

### Experimental validation of target gene repression by miRNAs

RNA was isolated from frozen cell pellets using the miRNeasy Mini Kit (Qiagen, Hilden, Germany) according to the manufacturer’s protocol. 500 ng total RNA were reverse transcribed in a 20 μl reaction utilizing oligo(dT)18 primer using the Transcriptor First Strand cDNA Synthesis Kit (Roche Applied Science, Mannheim, Germany). For qPCR analysis 2 μl 1:5 diluted cDNA were used in a 20 μl reaction using Power SYBR green PCR Mastermix (Applied Biosystems, Foster City, CA, USA). The used qPCR primers are listed in Supplementary Table 5. All primers were tested for specificity and efficacy. For all samples, three technical replicates were performed and relative expression was calculated with the ΔΔCt-method according to the manufacturer’s protocol. To identify suitable endogenous normalization control genes which were not altered by the tested miRNA, we revised the well-established house-keeping genes GAPDH, RPL19, TBP, HMBS, HPRT1, UBC and ACTB for potential miRNA binding-sites by utilizing the target gene prediction tools miRmap [90], TarBase [11], RNA22 [88], PicTar [86], TargetScan [89], miRanda [91], miRWalk [85] and PITA [87]. An overview over the tested house-keeping genes and identified binding sites with the corresponding prediction tool is presented in Supplementary Table 6. We considered a binding site predicted with at least one prediction tool as criterion for exclusion of the target gene to be used as a control. Due to the mutual exclusive binding site predictions of the tested control genes, choosing a unique control gene for all three miRNAs was not possible. Instead, we selected TBP as a control gene for miR-192 and miR-17 and UBC as a control gene for miR-200c. We considered a target gene with log2(2^-ΔΔCt) <=-0.5 to be effectively down-modulated by the miRNA. Upper and lower error bars were calculated as log2(2^-ΔΔCt) +/- ΔΔCt error propagation value.

### Western blot assay

Three days post transfection of fibroblast cells the supernatant was collected and used for detection of MMP2 protein levels by Western blot. Samples were heat denatured for 5 min at 96°C and loaded onto polyacrylamide gels (12%). After electro-transfer of separated protein extracts, nitrocellulose membranes were blocked with 5% BSA for 1h at RT. The primary antibody (MMP2 pAB ALX-210-753-R500, Enzo) was incubated at 4°C o/n in blocking solution. After extensive washing, membranes were incubated for 1h at RT with the corresponding secondary antibodies (anti-rabbit) coupled to horse reddish peroxidase. Protein signals were then detected with ECL-Prime reagent (GE Healthcare) and measurements were performed on a ChemiDoc XRS system (BioRad). Densitometric quantification of protein bands was performed using ImageLab software.

### Invasion assay

CCD-18Co cells were seeded in 6 well plates and grown to 80% confluency until they were transfected with 50 nM miRNA (mock, miR-192, miR-17 or miR-200c) and cultured for 3 days. The invasion assay was performed using 96 well Boyden-chamber plates (8 μm pore size, Corning, Big Flats, NY, USA), which were coated with 10 μg matrigel per well. Fibroblasts were harvested and 3 individually transfected wells were pooled for each condition. 1.5x105 CCD-18Co cells were seeded in outer wells of the Boyden-chamber in 150 μl DMEM medium supplemented with 10% FCS. HCT-116 colon cancer cells were seeded in the inner wells with a cell number of 5x104 in 50 μl per well. HCT-116 cells were kept in medium without serum. After 24 h incubation at 37°C invasion was measured. Cells were lysed with 1x cell dissociation solution (CDS) (Trevigen, Gaithersburg, MD, USA) supplemented with calcein-AM. HTS Transwell 96 well black receiver plates (Corning, Big Flats, NY, USA) were pre-incubated with PBS at 37°C. Meanwhile, the medium was removed from the outer wells of the Boyden-chamber plates. Washing of outer wells was performed very carefully by pipetting 150 μl PBS in and out. The PBS from the black receiver plate was discarded and 100 μl of 1x CDS-calcein was pipetted in each well. The inlay from the transwell plate was transferred carefully to the receiver plate avoiding any contacts. The plates were incubated for 1 h at 37°C and knocking at each side of the plate was performed every 15 min to ensure complete dissociation of cells attached to the lower side of the gel membrane. Finally, fluorescence was measured using 485 nm as excitation wavelength, 538 nm as the emission wavelength and integration of 60 ms.

### Proliferation assay

Fibroblast cells were transfected exactly as for the invasion assay. Three days post transfection the supernatant of transfected fibroblasts was collected. HCT-116 cells were seeded in 96 well plates with 5x10^4^ cells per well as for the invasion assay. After attachment of the colon cancer cells their medium was removed and replaced with the supernatant of the transfected fibroblasts. Proliferation of HCT-116 cells was measured with the XTT cell proliferation assay kit from PromoKine (PromoCell, Heidelberg, Germany) according to the manufacturer’s protocol. To test for significance reduction of proliferation, we applied one-way ANOVA Dunnett’s multiple comparison test using the multcomp [92] R-package with scrambled miRNA transfection as control (mimic control-1). Proliferation is presented as mean optical density (OD) at 450 nm absorbance +/- standard deviation.

## CONCLUSIONS

We identified miR-192, miR-17, miR-200c as miRNAs that down-regulate stromal target genes for ECM remodeling. Restoring miR-192, miR-17 and/or miR-200c expression in tumor stroma could reduce the invasive capacity of colon cancer cells. Our findings provide intriguing potential for therapeutic options, however, a final proof of the involvement of the proposed miRNAs and target genes in the pathogenesis of colorectal cancer requires further, in-depth investigations employing co-culture systems and animal models to pave the way for clinical applications.

## SUPPLEMENTARY MATERIALS FIGURES AND TABLES






















