# Supplementary material for: MiR-192, miR-200c and miR-17 are fibroblast-mediated inhibitors of colorectal cancer invasion
Source: Oncotarget. 2018 Oct 30;9(85):35559–80. doi: 10.18632/oncotarget.26263 (PMC6238973; doi:10.18632/oncotarget.26263)
Supplement: Supplementary file 1 [file oncotarget-09-35559-s001.pdf]

MiR-192, miR-200c and miR-17 are fibroblast-mediated inhibitors of colorectal cancer invasion

SUPPLEMENTARY MATERIALS

1 COMPUTATIONAL METHODS

1.1 The piecewise linear model

Using the miRNA expression values across all samples (K) we first defined q_1, q_2, q_3, q_4, q_5 and q_6 as the respective 0%, 20%, 40%, 60%, 80% and 100% quantiles. For every miRNA expression value (exp_k) of sample (k), the index of the next lowest quantile was determined as:

$$l_k^* := \min\{\operatorname{argmax}\{q_l <= exp_k\}, 5\}$$
$$\forall l \in \{1,...,6\} \qquad \text{(Equation 1)}$$

Next, parameters (z) were defined as:

$$z_{kl} = \begin{cases} \dfrac{q_{l_k^*+1} - exp_k}{q_{l_k^*+1} - q_{l_k^*}} & \text{if } l = l_k^* \\ \dfrac{exp_k - q_{l_k^*}}{q_{l_k^*+1} - q_{l_k^*}} & \text{if } l = l_k^* + 1 \\ 0 & \text{otherwise} \end{cases}$$
$$\forall l \in \{1,...,6\}$$

(Equation 2)

These z parameters form a special ordered set of type 2 (SOS2) [1], where at most two adjacent members can be non-zero and the sum of all members equal one. For every quantile, an additional parameter (β) was introduced to define the term (eff):

$$eff_k = \sum_{l=1}^{6} z_{kl} * \beta_l$$
$$\forall k \in K \qquad \text{(Equation 3)}$$

The z parameters are scalar and can be determined directly from the experimental data, whereas the β parameters are optimized by the solver. The term eff_k represents the predicted gene expression of sample k. Monotony is enforced by introducing a binary auxiliary variable (x) defined as:

$$x = \begin{cases} 1 & \text{if } \beta_{l+1} >= \beta_l \\ 0 & \text{if } \beta_{l+1} <= \beta_l \end{cases}$$
$$\forall l \in \{1,...,6\} \qquad \text{(Equation 4)}$$

Enforced model monotony was achieved by introducing additional constraints for the β parameters by means of a constant, M:

$$\beta_l \leq \beta_{l+1} + (1-x) * M$$
$$\beta_l \geq \beta_{l+1} - x * M$$
$$\forall l \in \{1,...,6\} \qquad \text{(Equation 5)}$$

with $M = 1000$ always fulfilling both inequalities in the corresponding dataset.

The optimization criterion of the model was to minimize the absolute difference between the measured gene expression (g_k)and the predicted gene expression (eff_k):

$$\min \sum_{k \in K} | eff_k - g_k | \qquad \text{(Equation 6)}$$

As this model is a minimization problem, the absolute value term can be solved by introducing an auxiliary variable (e_k) and transferring into two inequalities:

$$g_k - eff_k - e_k \leq 0$$
$$-g_k + eff_k - e_k \leq 0 \qquad \text{(Equation 7)}$$

The MILP solver Gurobi (http://www.gurobi.com) minimizes the sum of error terms across all samples by optimizing the β parameters within the constraints defined by equations 3 to 6. For each defined quantile of miRNA expression, a corresponding β coefficient is fitted against the measured gene expression for the target gene under investigation, yielding a step-wise approximation of target gene expression depending on miRNA expression.

Gene- and miRNA expression profiles were pre-processed and miRNA - target gene pairs were extracted from TarBase [2]. The piecewise linear model was established for every miRNA - target gene pair individually. The sample set was split into 5 equally sized partitions for 5-fold cross-validation, in which the parameters β and x were optimized in the model using samples from only 4 partitions (training partitions). The 5 β parameters were subsequently utilized to predict gene expression for the samples in the remaining fifth partition (validation partition). Predictive performance was estimated by calculating Pearson's correlation of measured and predicted gene expression across the validation partitions from all cross-validations. The binary parameter, x, indicates whether the model fit is strictly monotonically increasing or decreasing in the training partitions used. Only miRNA - target gene pairs

with predictive performances of r ≥ 0.25 and a strictly monotonically decreasing model fit for the majority of partitions (at least 3 out of 5) were considered for further analysis.

1.2 The linear model

In the linear model, the predicted expression (eff_k) of a gene in sample (k) was defined as:

$$eff_k = \beta_0 + exp_k * \beta \qquad \text{(Equation 8)}$$

in which the variable β and the offset variable β_0 were the optimization parameters and the variable exp_k represented the miRNA expression in sample k. The optimization criterion for the linear model was analogous to the piecewise linear model as described in the previous section. Gene- and miRNA expression data as well as miRNA - target gene pairs were utilized as described for the piecewise linear model. We performed 5-fold cross-validation by splitting the sample set in 5 equally sized partitions, ran the linear model using the samples of 4 partitions and evaluated the optimized parameters β_0 and β. Subsequently the parameters β_0 and β were utilized to predict the gene expression for the samples of the left-out fifth partition. The predictive performance was estimated by calculating Pearson's correlation of measured and predicted gene expression across validation samples of all cross-validations. Only miRNA - target gene pairs with predictive performances of r ≥ 0.25 were considered for further analysis.

1.3 Enrichment analysis in miRNA transfection experiments

We used normalized miRNA expression data as provided by the authors of the original studies. If miRNA transfection experiments were performed in replicates, we combined the expression values by using the median expression value per gene. If not directly provided in the dataset, we calculated the log ratio of gene expression of the test conditions (transfection with miRNA mimics or antagomiRs) and control conditions (mock or miRNA scrambled control) to observe differential gene expression. Enrichment tests were performed using the "geneSetTest" method from the limma package [3] depending on the experimental design: negative enrichment in case of miRNA over-expression (when using miRNA mimics) and positive enrichment in case of miRNA inhibition (when using antagomiRs). Due to the small number of experiments (n=41 for colon cancer, n=36 for prostate cancer), we refrained from multiple testing correction and treated p-values of the enrichment tests smaller or equal to 0.05 as significant. It is to note that a pre-miRNA measured in the transfection experiments under different experimental conditions could have been assigned to more than one stem-loop identifier in the TCGA datasets.

These stem-loop identifiers may show distinct expression profiles and share a highly overlapping, yet different set of target genes and therefore may have yielded different prediction results from the models. For instance, the pre-miRNA hsa-miR-7 matches 3 stem-loop identifiers used in the TCGA dataset: hsa-mir-7-1, hsa-mir-7-2 and hsa-mir-7-3. Therefore, we evaluated individual combinations of pre-miRNA and experimental condition from the transfection experiments and modeling data of the corresponding stem-loop miRNAs yielding 60 combinations for the colon- and 42 combinations for the prostate adenocarcinoma dataset.

1.4 Differential expression and gene set enrichment analysis of the predicted target genes

Identifying differentially expressed miRNA and genes

We tested all miRNAs and genes for differential expression in each molecular colorectal subgroup by a pairwise comparison to each of all other subgroups using a two-sided Student's *t*-test followed by multiple testing correction with the Benjamini-Hochberg method [4]. Any tested gene or miRNA with an adjusted p-value of at most 0.05 was considered to be differentially expressed. For each of the 4 subgroups, we selected genes and miRNAs which were differentially expressed between the subgroup under investigation and at least two other subgroups.

Gene set enrichment analysis

To elucidate the biological relevance of the predicted target genes in general and specifically in the molecular subgroups of colorectal cancer defined by Guinney *et al.* [5], we performed gene set enrichment analysis using TopGO [6]. TopGO functions as a wrapper package to facilitate enrichment analysis using gene set definitions from Gene Ontology (GO) [7]. We restrained our analysis to GO terms related to "biological process" and performed enrichment tests on the set of target genes predicted by the combined model for each miRNA individually. As global background we included all miRNA target genes provided by the TarBase database and chose "elim" as algorithm, "fisher" as the test statistics and tested only GO terms with a minimum number of 5 assigned genes neglecting very specific gene sets. Because multiple testing correction of p-values using standard techniques would come up with only very few significant gene sets and the tests performed by the "elim" method are not independent as described in the TopGO vignette (https://www.bioconductor.org/packages/release/bioc/vignettes/topGO/inst/doc/topGO.pdf, section 6.2), we set the significance threshold to a rather low value of 0.005 to reduce the false positive rate but did not correct for multiple testing. Typically, such enrichment tests yield large, difficult to manage list of gene sets. To overcome this, redundant GO terms were filtered using linear optimization. Redundancy between

two GO terms was quantified using Jaccard similarity coefficients. First, GO term-pairs with a high degree of overlap were included in the model. In the end, at most one GO term from a pair was chosen in such a way that the overall number of non-redundant GO terms was maximized. In addition, we assigned each term to one out of 18 broader categories according to their biological function: "angiogenesis", "apoptosis", "cell adhesion", "cell cycle", "cell differentiation", "cell migration", "developmental process", "DNA repair", "extracellular matrix process", "immune system process", "localization/transport", "metabolic process", "nervous system process", "receptor signaling", "response to stimuli", "transcription" and "translation". Terms that did not fit in any of those categories were assigned to the category "miscellaneous". To identify subgroup specific miRNAs and enriched gene sets, we filtered the identified gene sets having at least 3 genes being either up-regulated in the respective subgroup and the corresponding miRNA being down-regulated in the same molecular subgroup or *vice versa*.

1.5 Functional grouping of genes related to extracellular matrix remodeling

According to their molecular function in the context of extracellular matrix (ECM) remodeling, candidate genes were grouped into one of the following categories: "ECM component", "ECM degradation", "ECM synthesis", "ECM - cell signaling" and "Others". Genes that code for proteins assembling essential parts of ECM structures like collagen, laminin or fibrillin were assigned to the category "ECM component". "ECM degradation" summarizes candidate genes like matrix metallopeptidases that are directly or indirectly involved in ECM decay. Target genes that are involved in the synthesis of ECM structures or maintain its integrity like lysyl oxidase were assigned to the category "ECM synthesis". Finally, all genes that are part of direct ECM - cell interactions like integrins or are involved in ECM - cell communication like TGF-β were assigned to the category "ECM - cell signaling". Genes that did not clearly fit in one of the above categories were combined in the category "Others". It is to note that even though genes could functionally belong to more than one group, we restricted their functional grouping to the most relevant category according to the literature.

1.6 Analysis of tumor- and stromal cell content

Based on the clinical data from the TCGA colon adenocarcinoma dataset provided by the TCGA consortium [8], we analyzed tumor- and stromal cell content among the defined molecular subgroups. In detail, we used the attributes "percent_stromal_cells_BOTTOM", "percent_stromal_cells_TOP", "percent_tumor_cells_BOTTOM" and "percent_tumor_cells_TOP", all of which were determined by histopathologists after individual image analysis. "BOTTOM" and "TOP" refer to the bottom and top section of the tissue which has been imaged. We compared samples of one molecular colorectal subgroup with the combination of all samples from all other molecular subgroups and performed a significance test using a two-sided Student's *t*-test.

1.7 Correlation analysis of miRNA expression and target gene protein expression

We tested the identified miRNA – target gene pairs for potential inverse correlation of miRNA expression and protein abundance. We employed a protein data set generated by the Clinical Proteomic Tumour Analysis Consortium (CPTAC) [9] comprising quantification of 5561 proteins in 95 colon and rectal samples from the TCGA cohort whereof 49 samples were present in the TCGA colon miRNA expression data set. Normalized spectral counts were used for protein. We computed Pearson's correlation of miRNA expression and protein spectral counts for all miRNA – target gene pairs where the data was available. Statistical significance for each miRNA – target gene pair was calculated using a rank-based permutation test (n = 1,000,000). Briefly, for both data matrices the sample labels as well as the feature labels were shuffled and the Pearson' correlation coefficient was calculated for each miRNA – target gene pair. This process was repeated 1,000,000 times.

2 RESULTS

2.1 Evaluating model predictions using miRNA transfection experiments

TCGA colon adenocarcinoma dataset

To confirm predictions and evaluate both models, we assembled expression data from 60 miRNA or antagomiR transfection experiments from the Gene Expression Omnibus (GEO), and tested for significant enrichment of the predicted target genes of the respective miRNA among the genes that were down-regulated by miRNA transfection (details in Supplementary 1.3 and workflow in Supplementary Figure 1). The piecewise linear model predicted miRNA target genes, which had better enrichment in experimental datasets from 26 transfection experiments, whereas the linear model predicted miRNA target genes, for which enrichment was only better in 20 experimental datasets. Combining both the piecewise linear and linear models using the union of predicted target genes, improved enrichment in 43 experimental datasets above that achieved by either model alone. No miRNA targets predicted by either model or the combination of both were significantly enriched in datasets from 14 experiments. Supplementary Table 7A gives an overview of these results together with the accession numbers from the datasets in the GEO database, used cell lines, transfected miRNAs, conditions and the p-values for each model and the combination of both models.

TCGA prostate adenocarcinoma dataset

We performed enrichment tests on target genes that were predicted by the linear model, the piecewise linear model and the combination of both using the TCGA prostate adenocarcinoma dataset. We investigated publicly available expression datasets of 42 miRNA transfection experiments which were performed using prostate cancer cell lines. When comparing the linear and the piecewise linear model, 10 experiments had better enrichments using the piecewise linear model, 8 experiments had better enrichments using the linear model and no significant enrichments were observed in the remaining 24 experiments. The combination of both methods showed superiority over the linear and piecewise linear model: we observed better enrichment among 12 experiments when combining the models whereas for the linear and the piecewise linear model we observed no respective 3 experiments with lower p-values than the model combination. There were 3 experiments with equal significance levels and 24 experiments with no significant enrichment in any model. Detailed results are given in Supplementary Table 7B.

2.2 Investigating the biological relevance of target gene predictions

To benchmark the model predictions for biological relevance, we performed gene set enrichment analysis using the predicted target genes for each miRNA from either the linear or the combined model, both performed using the TCGA colon adenocarcinoma dataset. Target gene predictions from the combined model identified 212 significantly enriched gene sets beyond those identified using the linear model alone. Among these additionally identified gene sets were “transforming growth factor beta receptor signaling pathway”, “positive regulation of epithelial cell migration” and “endothelial cell migration” as potentially regulated by miR-29b-2 which has been shown in breast cancer [10], prostate cancer [11] and multiple myeloma [12].

2.3 Functional relevance of experimentally validated target genes

We briefly describe the results of our literature analysis concerning the functional relevance (in the context of extracellular matrix remodeling, tumor development and progression, metastasis and clinical outcome) of the experimentally validated target genes in colorectal cancer and other tumor entities.

2.3.1 Validated target genes of miR-192

FBN1 is an extracellular glycoprotein secreted by fibroblasts that forms long, elastic microfibrils as important components of the extracellular matrix. It has been shown to promote ovarian tumorigenesis and metastasis in mouse models [13]. When highly expressed, FBN1 indicates poor overall survival in ovarian cancer [13]. Inhibition of E-cadherin and β-catenin and stimulation of matrix metallopeptidase 2, 9 and 13 are further characteristics of FBN1 as described by Wang *et al.* [13]. PLOD1 is another important collagen crosslinking enzyme necessary for the biogenesis and stability of collagens [14]. Gilkes and colleagues reported an increased expression of PLOD1 and another gene family member, PLOD2, in breast cancer tissue compared to normal breast tissue [14]. The cell surface peroxidase PXDN is, when secreted into the extracellular space, involved in ECM formation by crosslinking collagen IV. It has been shown that silencing of PXDN leads to significant reduction of cancer cell invasion in melanoma [15]. The growth factor FGF2 is part of many cellular processes and particularly known as an inducer of angiogenesis. Knuchel and co-workers identified fibroblast-bound FGF2 as part of the FGFR-SRC- ITGAV/ITGB5 cascade that induces tumor cell adhesion to fibroblasts and tumor cell motility which was shown in 2D and 3D models of colorectal cancer cells [16]. Interestingly, Musumeci and co-workers showed an direct relation between the down-regulation of miR-15 and miR-16 and the up-regulation of their direct target genes FGF2 and FGFR1 in tumor-associated stroma cells of prostate cancer [17]. The linker protein DST is a component of hemidesmosomes [18] and connects intermediate filaments to the actin cytoskeleton and helps to form actin bundles around the nucleus.

2.3.2 Validated target genes of miR-200c

In leiomyoma, a benign smooth muscle cell tumor occurring in the uterus, TIMP2 and FBLN5 were identified as direct targets of miR-200c [19]. The extracellular glycoprotein FBLN5 is involved in the formation of elastic fibers and antagonizes fibronectin-mediated signaling by binding to the same integrin receptors (reviewed in [20]). FBLN5 was found to be induced by TGF-β signaling in fibroblasts and endothelial cells [21] and plays a role in initiating and enhancing epithelial-to-mesenchymal transition in normal and malignant mammary epithelial cells [22]. In the same study, FBLN5 was shown to promote the expression and activity of MMP2 and MMP9 and the growth of human 4T1 breast cancer cells in mice [22]. Contrary to its tumor promoting role, it was observed that FBLN5 can suppress the formation of metastasis in lung and liver [23]. The glycoprotein FN1 was found to be significantly down-regulated after re-expression of miR-200c in human Hec50 endometrial cancer cells [24]. By binding to the α5β1 integrin transmembrane receptor dimer, FN1 mediates cell-matrix adhesion and is directly involved in cell migration and invasion [25]. Overexpression of FN1 was observed in myofibroblasts and in epithelial cells in samples of colorectal cancer patients [26]. Elevated epithelial expression of FN1 can be an indicator of lymph node metastasis in primary colorectal cancers [27]. SERPINH1 (also referred as HSP47) is involved in the synthesis and deposition of collagen as a chaperone present in the endoplasmatic reticulum. SERPINH1 is linked to the progression of

breast cancer [28], cell migration and invasion of cancer cells in cervical squamous cell carcinoma [29], and tumor growth and invasion in glioma [30]. The transcription factor ETS1 plays a role in numerous tumor types and is linked to tumor progression, invasion, metastasis and angiogenesis. It is active in cancer cells, cancer-associated fibroblasts and endothelial cells (reviewed in [31]). In particular in colorectal cancer, stromal protein levels of ETS1 were significantly associated with the formation of lung metastasis [32]. It was reported to be a direct target of miR-200c in mouse embryonic stem cells [33]. Of note, ETS1 is known to repress the transcription of the miR-192 host gene [34] and can induce the expression of KDR, another potential target gene of miR-200c.

2.3.3 Validated target genes of miR-17

The cytokine transforming growth factor beta 1 (TGFB1) is a well-known ligand and driver of TGF-β signaling which is central for tumor development and progression, angiogenesis, epithelial-to-mesenchymal transition and metastasis (reviewed in [35–37]). Tumor cell-derived TGF-β is actively promoting the transdifferentiation of fibroblasts to a myofibroblast- or tumor-associated fibroblast phenotype [38]. In the course of colorectal cancer progression, the interaction of tumor cells and tumor-associated fibroblasts leads to a hyperactivation of TGF-β signaling in tumor-associated fibroblasts and an elevated secretion of TGF-β into the extracellular space whereas TGF-β signaling is lost in most cancer cells [39]. Besides other known target genes, TGF-β is known to increase its own expression [39] and the expression of MMP2 [40]. LAMC1 is a member of a large family of extracellular glycoproteins which form large polymers with other laminin isoforms and form a major constituent of the basement membrane besides collagen. LAMC1 is essential during embryonic development [41]. In the context of tumor development, it is known that LAMC1 promotes cancer cell migration and invasion in prostate cancer [11] and enhances tumor cell motility and invasion in uterine and ovarian carcinomas [42]. Furthermore, it was shown that LAMC1 is a direct target of miR-22 and miR-29a/b/c in prostate cancer [11, 43] and of miR-205 in receptor triple-negative breast cancer [44].

REFERENCES

1. Beale EML, Tomlin JA. Special facilities in a general mathematical programming system for non-convex problems using ordered sets of variables. Oper Res. 1970; 69:447–54.
2. Vlachos IS, Paraskevopoulou MD, Karagkouni D, Georgakilas G, Vergoulis T, Kanellos I, Anastasopoulos IL, Maniou S, Karathanou K, Kalfakakou D, Fevgas A, Dalamagas T, Hatzigeorgiou AG. DIANA-TarBase v7.0: indexing more than half a million experimentally supported miRNA:mRNA interactions. Nucleic Acids Res. 2015; 43:D153–9. https://doi.org/10.1093/nar/gku1215.
3. Goeman JJ, Buhlmann P. Analyzing gene expression data in terms of gene sets: methodological issues. Bioinformatics. 2007; 23:980–7. https://doi.org/10.1093/bioinformatics/btm051.
4. Benjamini Y, Hochberg Y. Controlling the false discovery rate: a practical and powerful approach to multiple testing. J R Stat Soc Ser B. 1995; 57:289–300. https://doi.org/10.2307/2346101.
5. Guinney J, Dienstmann R, Wang X, de Reyniès A, Schlicker A, Soneson C, Marisa L, Roepman P, Nyamundanda G, Angelino P, Bot BM, Morris JS, Simon IM, et al. The consensus molecular subtypes of colorectal cancer. Nat Med. 2015; 21:1350–6. https://doi.org/10.1038/nm.3967.
6. Alexa A, Rahnenfuhrer J, Lengauer T. Improved scoring of functional groups from gene expression data by decorrelating GO graph structure. Bioinformatics. 2006; 22:1600–7. https://doi.org/10.1093/bioinformatics/btl140.
7. Ashburner M, Ball CA, Blake JA, Butler H, Cherry JM, Eppig JT, Harris M, Hill DP, Mungall C, Reiser L, Rhee S, Richardson JE, Richter J, et al. Creating the gene ontology resource: design and implementation. Genome Res. 2001; 11:1425–33. https://doi.org/10.1101/gr.180801.
8. Cancer Genome Atlas Network TCGA. Comprehensive molecular characterization of human colon and rectal cancer. Nature. 2012; 487:330–7. https://doi.org/10.1038/nature11252.
9. Zhang B, Wang J, Wang X, Zhu J, Liu Q, Shi Z, Chambers MC, Zimmerman LJ, Shaddox KF, Kim S, Davies SR, Wang S, Wang P, et al. Proteogenomic characterization of human colon and rectal cancer. Nature. 2014; 513:382–7. https://doi.org/10.1038/nature13438.
10. Chou J, Lin JH, Brenot A, Kim J, Provot S, Werb Z. GATA3 suppresses metastasis and modulates the tumour microenvironment by regulating microRNA-29b expression. Nat Cell Biol. 2013; 15:201–13. https://doi.org/10.1038/ncb2672.
11. Nishikawa R, Goto Y, Kojima S, Enokida H, Chiyomaru T, Kinoshita T, Sakamoto S, Fuse M, Nakagawa M, Naya Y, Ichikawa T, Seki N. Tumor-suppressive microRNA-29s inhibit cancer cell migration and invasion via targeting LAMC1 in prostate cancer. Int J Oncol. 2014; 45:401–10. https://doi.org/10.3892/ijo.2014.2437.
12. Amodio N, Bellizzi D, Leotta M, Raimondi L, Biamonte L, D'Aquila P, Di Martino MT, Calimeri T, Rossi M, Lionetti M, Leone E, Passarino G, Neri A, et al. miR-29b induces SOCS-1 expression by promoter demethylation and negatively regulates migration of multiple myeloma and endothelial cells. Cell Cycle. 2013; 12:3650–62. https://doi.org/10.4161/cc.26585.
13. Wang Z, Liu Y, Lu L, Yang L, Yin S, Wang Y, Qi Z, Meng J, Zang R, Yang G. Fibrillin-1, induced by Aurora-A but inhibited by BRCA2, promotes ovarian cancer metastasis. Oncotarget. 2015; 6:6670–83. https://doi.org/10.18632/oncotarget.3118.

14. Gilkes DM, Bajpai S, Wong CC, Chaturvedi P, Hubbi ME, Wirtz D, Semenza GL. Procollagen Lysyl Hydroxylase 2 is essential for hypoxia-induced breast cancer metastasis. Mol Cancer Res. 2013; 11:456–66. https://doi.org/10.1158/1541-7786.MCR-12-0629.

15. Jayachandran A, Prithviraj P, Lo PH, Walkiewicz M, Anaka M, Woods BL, Tan B, Behren A, Cebon J, McKeown SJ. Identifying and targeting determinants of melanoma cellular invasion. Oncotarget. 2016; 7:41186–202. https://doi.org/10.18632/oncotarget.9227.

16. Knuchel S, Anderle P, Werfelli P, Diamantis E, Rüegg C. Fibroblast surface-associated FGF-2 promotes contact-dependent colorectal cancer cell migration and invasion through FGFR-SRC signaling and integrin αvβ5-mediated adhesion. Oncotarget. 2015; 6:14300–17. https://doi.org/10.18632/oncotarget.3883.

17. Musumeci M, Coppola V, Addario A, Patrizii M, Maugeri-Saccà M, Memeo L, Colarossi C, Francescangeli F, Biffoni M, Collura D, Giacobbe A, D'Urso L, Falchi M, et al. Control of tumor and microenvironment cross-talk by miR-15a and miR-16 in prostate cancer. Oncogene. 2011; 30:4231–42. https://doi.org/10.1038/onc.2011.140.

18. Sonnenberg A, Liem RK. Plakins in development and disease. Exp Cell Res. 2007; 313:2189–203. https://doi.org/10.1016/j.yexcr.2007.03.039.

19. Chuang TD, Panda H, Luo X, Chegini N. miR-200c is aberrantly expressed in leiomyomas in an ethnic-dependent manner and targets ZEBs, VEGFA, TIMP2, and FBLN5. Endocr Relat Cancer. 2012; 19:541–56. https://doi.org/10.1530/ERC-12-0007.

20. Yanagisawa H, Schluterman MK, Brekken RA. Fibulin-5, an integrin-binding matricellular protein: its function in development and disease. J Cell Commun Signal. 2009; 3:337–47. https://doi.org/10.1007/s12079-009-0065-3.

21. Schiemann WP, Blobe GC, Kalume DE, Pandey A, Lodish HF. Context-specific effects of fibulin-5 (DANCE/EVEC) on cell proliferation, motility, and invasion. Fibulin-5 is induced by transforming growth factor-beta and affects protein kinase cascades. J Biol Chem. 2002; 277:27367–77. https://doi.org/10.1074/jbc.M200148200.

22. Lee YH, Albig AR, Regner M, Schiemann BJ, Schiemann WP. Fibulin-5 initiates epithelial-mesenchymal transition (EMT) and enhances EMT induced by TGF-beta in mammary epithelial cells via a MMP-dependent mechanism. Carcinogenesis. 2008; 29:2243–51. https://doi.org/10.1093/carcin/bgn199.

23. Devitt Moller H, Ralfkjaer U, Cremers N, Frankel M, Troelsgaard Pedersen R, Klingelhofer J, Yanagisawa H, Grigorian M, Guldberg P, Sleeman J, Lukanidin E, Ambartsumian N. Role of Fibulin-5 in metastatic organ colonization. Mol Cancer Res. 2011; 9:553–63. https://doi.org/10.1158/1541-7786.MCR-11-0093.

24. Cochrane DR, Spoelstra NS, Howe EN, Nordeen SK, Richer JK. MicroRNA-200c mitigates invasiveness and restores sensitivity to microtubule-targeting chemotherapeutic agents. Mol Cancer Ther. 2009; 8:1055–66. https://doi.org/10.1158/1535-7163.MCT-08-1046.

25. Viana Lde S, Affonso RJ Jr, Silva SR, Denadai MV, Matos D, Salinas de Souza C, Waisberg J. Relationship between the Expression of the extracellular matrix genes SPARC, SPP1, FN1, ITGA5 and ITGAV and clinicopathological parameters of tumor progression and colorectal cancer dissemination. Oncology. 2013; 84:81–91. https://doi.org/10.1159/000343436.

26. Hanamura N, Yoshida T, Matsumoto E, Kawarada Y, Sakakura T. Expression of fibronectin and tenascin-C mRNA by myofibroblasts, vascular cells and epithelial cells in human colon adenomas and carcinomas. Int J Cancer. 1997; 73:10–5.

27. Meeh PF, Farrell CL, Croshaw R, Crimm H, Miller SK, Oroian D, Kowli S, Zhu J, Carver W, Wu W, Pena E, Buckhaults PJ. A gene expression classifier of node-positive colorectal cancer. Neoplasia. 2009; 11:1074–83.

28. Zhu J, Xiong G, Fu H, Evers BM, Zhou BP, Xu R. Chaperone Hsp47 Drives malignant growth and invasion by modulating an ECM gene network. Cancer Res. 2015; 75:1580–91. https://doi.org/10.1158/0008-5472.CAN-14-1027.

29. Seki N, Kinoshita T, Nohata N, Yoshino H, Itesako T, Fujimura L, Mitsuhashi A, Usui H, Enokida H, Nakagawa M, Shozu M, Seki N. Tumor-suppressive microRNA-29a inhibits cancer cell migration and invasion via targeting HSP47 in cervical squamous cell carcinoma. Int J Oncol. 2013; 43:1855–63. https://doi.org/10.3892/ijo.2013.2145.

30. Zhao D, Jiang X, Yao C, Zhang L, Liu H, Xia H, Wang Y. Heat shock protein 47 regulated by miR-29a to enhance glioma tumor growth and invasion. J Neurooncol. 2014; 118:39–47. https://doi.org/10.1007/s11060-014-1412-7.

31. Dittmer J. The role of the transcription factor Ets1 in carcinoma. Semin Cancer Biol. 2015; 35:20–38. https://doi.org/10.1016/j.semcancer.2015.09.010.

32. Sato T, Miwa A. Ets-1 and integrin beta3 for lung metastasis from colorectal cancer. APMIS. 2002; 110:347–53.

33. Gill JG, Langer EM, Lindsley RC, Cai M, Murphy TL, Murphy KM. Snail promotes the cell-autonomous generation of Flk1(+) endothelial cells through the repression of the microRNA-200 family. Stem Cells Dev. 2012; 21:167–76.

34. Dong Z. Acetylation of Ets-1 is the key to chromatin remodeling for miR-192 expression. Sci Signal. 2013; 6:pe21–pe21. https://doi.org/10.1126/scisignal.2004299.

35. Ikushima H, Miyazono K. TGFβ signalling: a complex web in cancer progression. Nat Rev Cancer. 2010; 10:415–24. https://doi.org/10.1038/nrc2853.

36. Pickup M, Novitskiy S, Moses HL. The roles of TGFβ in the tumour microenvironment. Nat Rev Cancer. 2013; 13:788–99. https://doi.org/10.1038/nrc3603.

37. Hawinkels LJ, Paauwe M, Verspaget HW, Wiercinska E, van der Zon JM, van der Ploeg K, Koelink PJ, Lindeman JH, Mesker W, ten Dijke P, Sier CF. TGF-β signalling and its role in cancer progression and

metastasis. Cancer Metastasis Rev. 2012; 31:553–68. https://doi.org/10.1007/s10555-012-9375-7.

38. Lewis MP, Lygoe KA, Nystrom ML, Anderson WP, Speight PM, Marshall JF, Thomas GJ. Tumour-derived TGF-β1 modulates myofibroblast differentiation and promotes HGF/SF-dependent invasion of squamous carcinoma cells. Br J Cancer. 2004; 90:822–32. https://doi.org/10.1038/sj.bjc.6601611.

39. Hawinkels LJAC, Paauwe M, Verspaget HW, Wiercinska E, van der Zon JM, van der Ploeg K, Koelink PJ, Lindeman JHN, Mesker W, ten Dijke P, Sier CFM. Interaction with colon cancer cells hyperactivates TGF-β signaling in cancer-associated fibroblasts. Oncogene. 2014; 33:97–107. https://doi.org/10.1038/onc.2012.536.

40. Itoh H, Kishore AH, Lindqvist A, Rogers DE, Word RA. Transforming growth factor β1 (TGFβ1) and progesterone regulate matrix metalloproteinases (MMP) in human endometrial stromal cells. J Clin Endocrinol Metab. 2012; 97:E888–97. https://doi.org/10.1210/jc.2011-3073.

41. Schéele S, Nyström A, Durbeej M, Talts JF, Ekblom M, Ekblom P. Laminin isoforms in development and disease. J Mol Med. 2007; 85:825–36. https://doi.org/10.1007/s00109-007-0182-5.

42. Kashima H, Wu RC, Wang Y, Sinno AK, Miyamoto T, Shiozawa T, Wang TL, Fader AN, Shih IM. Laminin C1 expression by uterine carcinoma cells is associated with tumor progression. Gynecol Oncol. 2015; 139:338–44. https://doi.org/10.1016/j.ygyno.2015.08.025.

43. Pasqualini L, Bu H, Puhr M, Narisu N, Rainer J, Schlick B, Schäfer G, Angelova M, Trajanoski Z, Börno ST, Schweiger MR, Fuchsberger C, Klocker H. miR-22 and miR-29a are members of the androgen receptor cistrome modulating LAMC1 and Mcl-1 in prostate cancer. Mol Endocrinol. 2015; 29:1037–54. https://doi.org/10.1210/me.2014-1358.

44. Piovan C, Palmieri D, Di Leva G, Braccioli L, Casalini P, Nuovo G, Tortoreto M, Sasso M, Plantamura I, Triulzi T, Taccioli C, Tagliabue E, Iorio M V, et al. Oncosuppressive role of p53-induced miR-205 in triple negative breast cancer. Mol Oncol. 2012; 6:458–72. https://doi.org/10.1016/j.molonc.2012.03.003.

45. Koo BH, Kim YH, Han JH, Kim DS. Dimerization of matrix metalloproteinase-2 (MMP-2). J Biol Chem. 2012; 287:22643–53. https://doi.org/10.1074/jbc.M111.337949.

46. Christensen J, El-Gebali S, Natoli M, Sengstag T, Delorenzi M, Bentz S, Bouzourene H, Rumbo M, Felsani A, Siissalo S, Hirvonen J, Vila MR, Saletti P, et al. Defining new criteria for selection of cell-based intestinal models using publicly available databases. BMC Genomics. 2012; 13:274. https://doi.org/10.1186/1471-2164-13-274.

47. Calon A, Espinet E, Palomo-Ponce S, Tauriello DVF, Iglesias M, Céspedes MV, Sevillano M, Nadal C, Jung P, Zhang XHF, Byrom D, Riera A, Rossell D, et al. Dependency of colorectal cancer on a TGF-β-driven program in stromal cells for metastasis initiation. Cancer Cell. 2012; 22:571–84. https://doi.org/10.1016/j.ccr.2012.08.013.

48. Nishida N, Nagahara M, Sato T, Mimori K, Sudo T, Tanaka F, Shibata K, Ishii H, Sugihara K, Doki Y, Mori M. Microarray analysis of colorectal cancer stromal tissue reveals upregulation of two oncogenic miRNA clusters. Clin Cancer Res. 2012; 18:3054–70. https://doi.org/10.1158/1078-0432.CCR-11-1078.

49. Della Vittoria Scarpati G, Calura E, Di Marino M, Romualdi C, Beltrame L, Malapelle U, Troncone G, De Stefano A, Pepe S, De Placido S, D'Incalci M, Marchini S, Carlomagno C. Analysis of differential miRNA expression in primary tumor and stroma of colorectal cancer patients. Biomed Res Int. 2014; 2014:840921. https://doi.org/10.1155/2014/840921.

50. Kozomara A, Griffiths-Jones S. miRBase: annotating high confidence microRNAs using deep sequencing data. Nucleic Acids Res. 2014; 42:D68–73. https://doi.org/10.1093/nar/gkt1181.

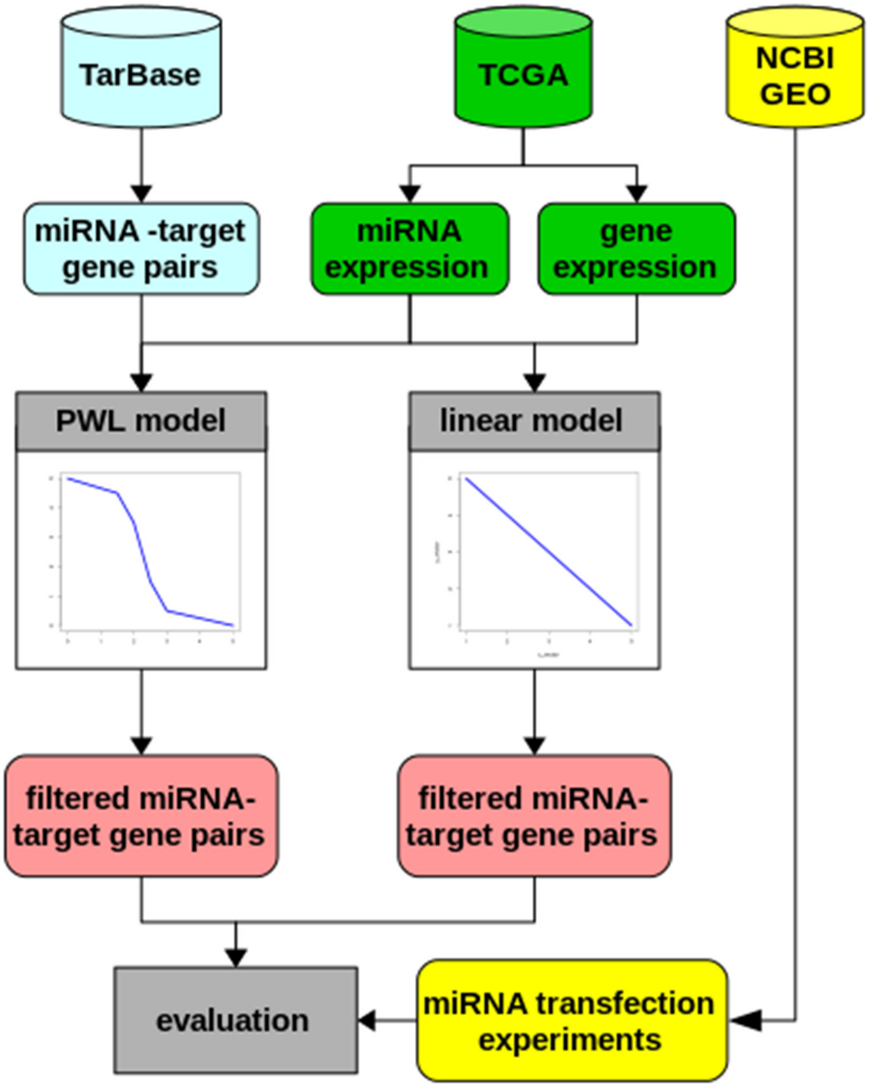

Supplementary Figure 1: Workflow to set up and compare the piecewise linear model with the linear model. MiRNA-target gene pairs were extracted from the database TarBase, gene- and miRNA expression data were taken from TCGA. For every selected miRNA-gene pair, a piecewise linear model and a linear model were set up and solved. To confirm the predicted target genes and to compare the performance of both models, we assembled expression data from miRNA transfection experiments and tested the predicted target genes from the models for significant enrichment among the down-regulated target genes from the transfection experiments. (TCGA = The Cancer Genome Atlas; PWL model = piecewise linear model; NCBI GEO = NCBI Gene expression Omnibus).

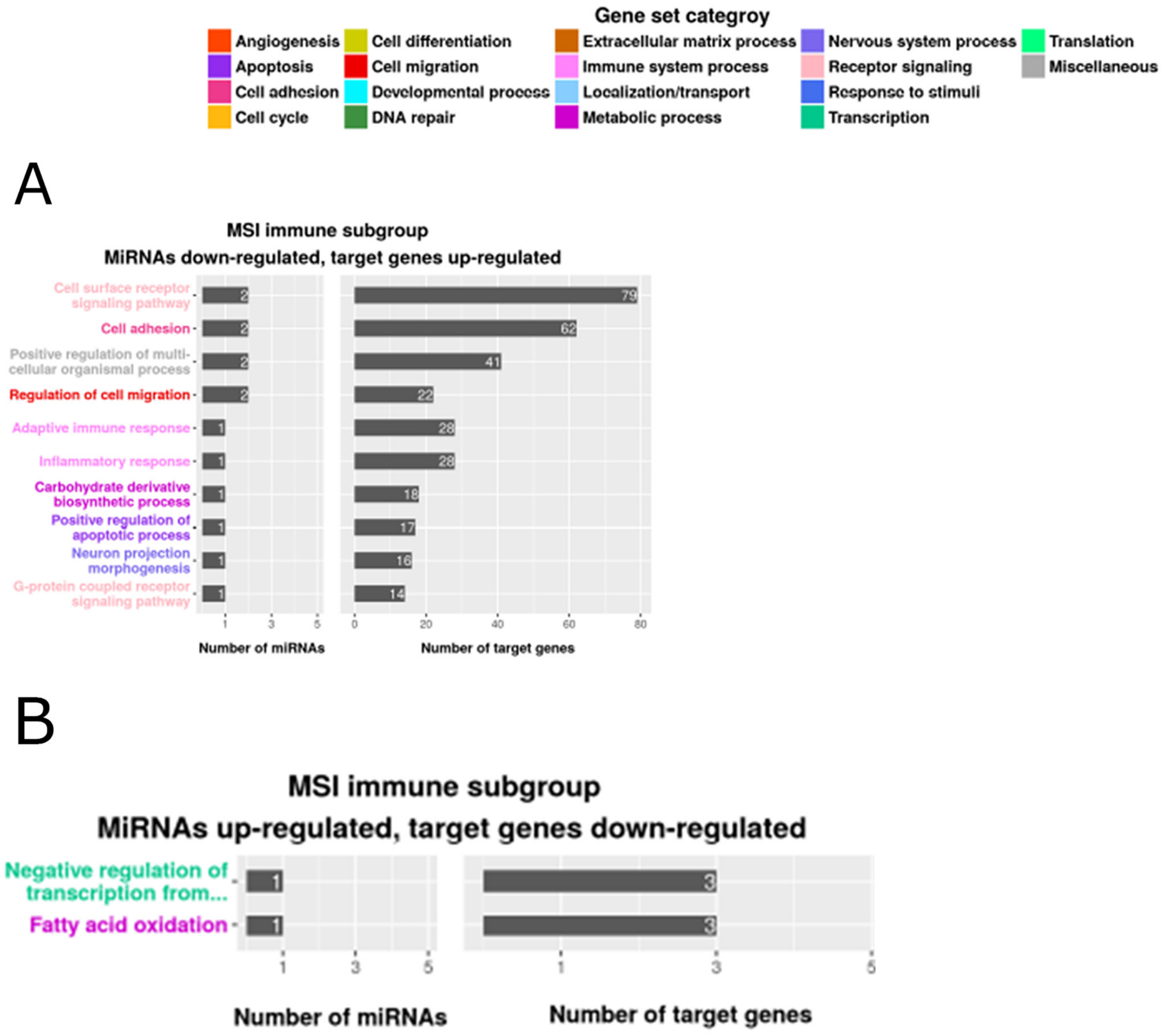

Supplementary Figure 2: Enriched gene sets of differentially expressed genes and miRNAs in the MSI immune-, the canonical- and the metabolic subgroup. Top 10 gene sets listed according to the number of regulating miRNAs (left) and the number of enriched target genes (right) are shown. The color indicates the assigned gene set category. **(A)** MSI immune subgroup, miRNAs down-regulated, target genes up-regulated. **(B)** MSI immune subgroup, miRNAs up-regulated, target genes down-regulated.

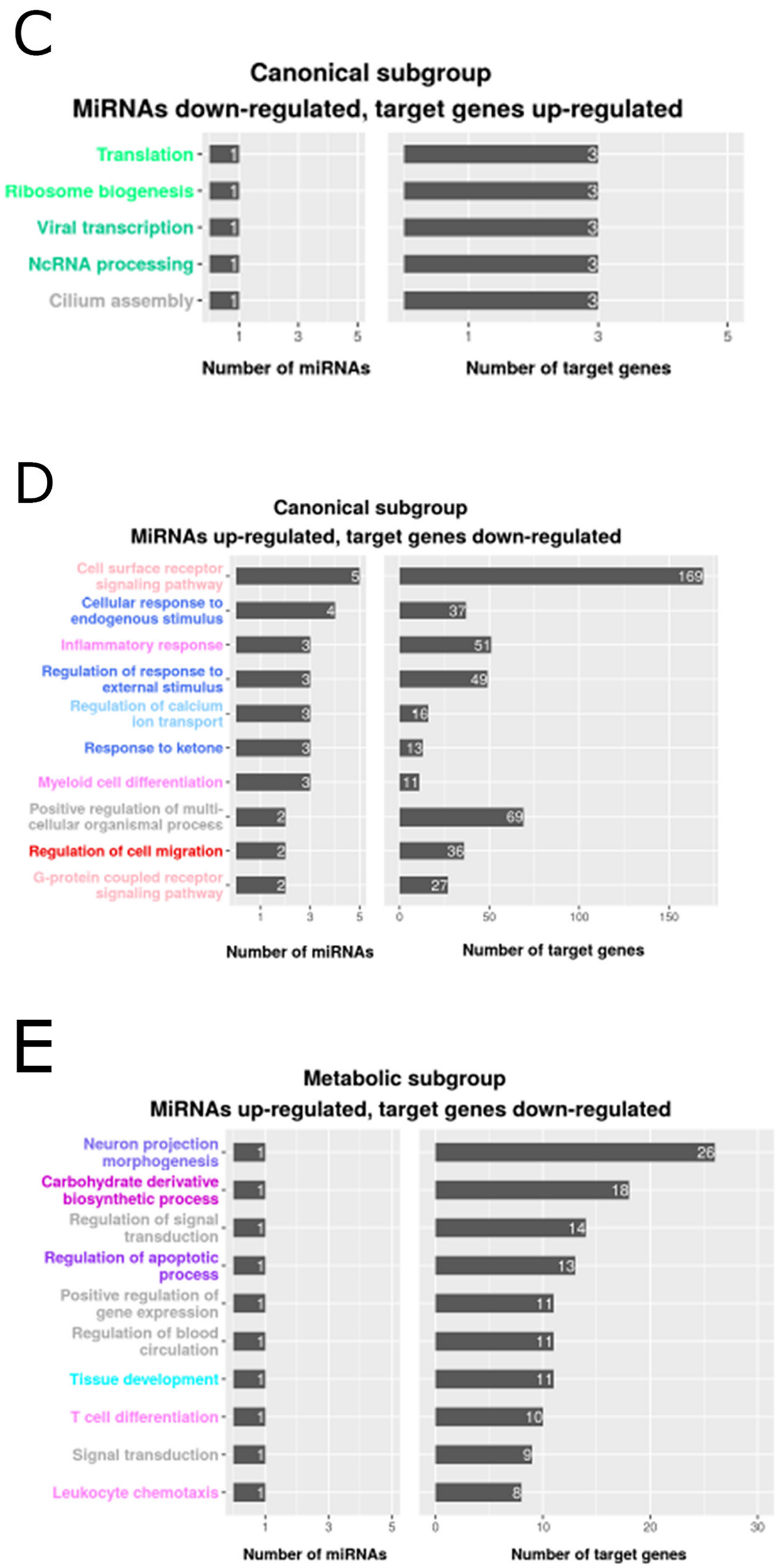

Supplementary Figure 2: (Continued) Enriched gene sets of differentially expressed genes and miRNAs in the MSI immune-, the canonical- and the metabolic subgroup. (C) Canonical subgroup, miRNAs down-regulated, target genes up-regulated. **(D)** Canonical subgroup, miRNAs up-regulated, target genes down-regulated. **(E)** Metabolic subgroup, miRNAs up-regulated, target genes down-regulated.

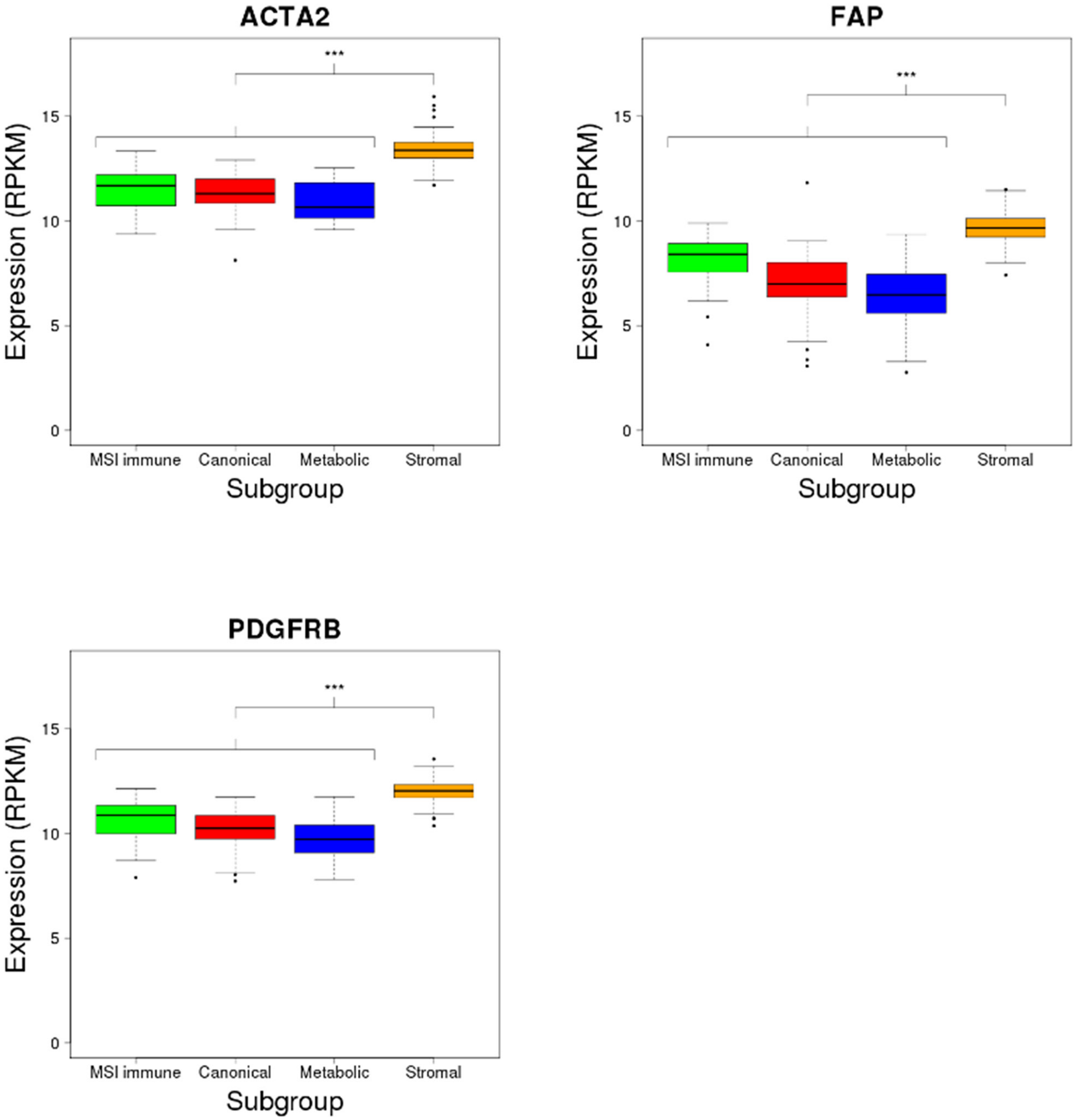

Supplementary Figure 3: Box-plots of expression values of tumor-associated fibroblast marker genes across molecular subgroups. ACTA2, FAP and PDGFRB are typical marker genes for tumor-associated fibroblasts. All of them were significantly up-regulated in the stromal subgroup in the TCGA colon adenocarcinoma dataset. Significance values were determined with Student's *t*-test and multiple testing corrected using the method by Benjamini-Hochberg, p=2.34e-32 for ACTA2, p=1.32e-11 for FAP and p=3.37e-21 for PDGFRB, (***) represents p-values below 0.001.

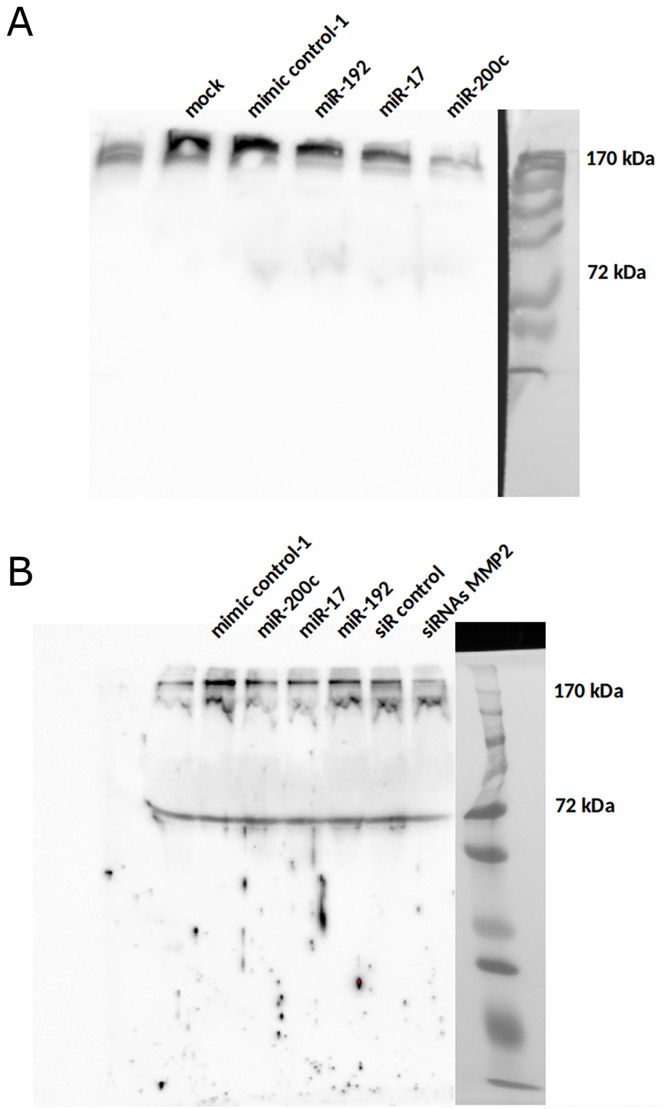

Supplementary Figure 4: Western blot analysis of MMP2 protein level in miRNA transfected fibroblasts. Western blot assays to detect the relative MMP2 protein abundance after transfection with miR-200c, miR-17 and miR-192 in **(A)** the supernatant of CCD-18Co cells (n=1) and **(B)** in the supernatant of MRC5 cells (n=1). In addition, MRC5 cells were also transfected with a specific siRNA targeting MMP2. We observed two bands in the western blots, indicating the detection of pro-MMP2 at ~72 kDa and an MMP2 dimer complex at ~170 kDa. Known MMP2 protein isoforms are described in [45]. The signal intensity from the 170kDa band was used to calculate the relative abundances. After siRNA-mediated knockdown of MMP2 in MRC5 cells, we measured a relative protein abundance of 0.46 using a siRNA mimic control as a reference, which confirms the selected band and the specificity of the used antibody. We used a mimic control transfection as a reference and observed a relative MMP2 protein abundance of 0.57, 0.54 and 0.62 for miR-200c, miR-17 and miR-192, respectively, in MRC5 cells (n=1). In CCD-18Co cells, we detected a relative protein abundance of 0.83, 0.68 and 0.29 for miR-192, miR-17 and miR-200c after transfection (n=1).

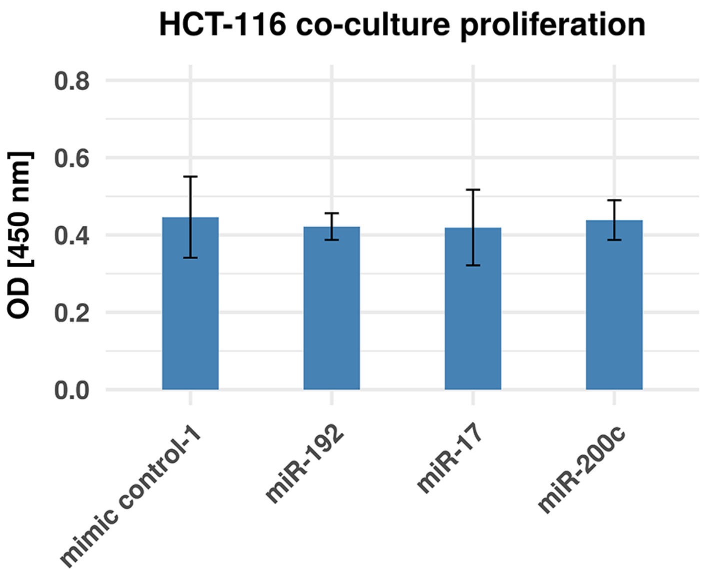

Supplementary Figure 5: Proliferation rates of HCT-116 under co-culture conditions. Proliferation rates are presented as optical density (OD) at 450 nm absorbance measured 24h post transfection. HCT-116 cells were co-cultured with the supernatant of CCD-18Co cells which were transfected with miR-192, miR-17, miR-200c and a mimic control beforehand. One-way ANOVA Dunnett's multiple comparison test using mimic control-1 as a reference revealed no significant differences in either condition.

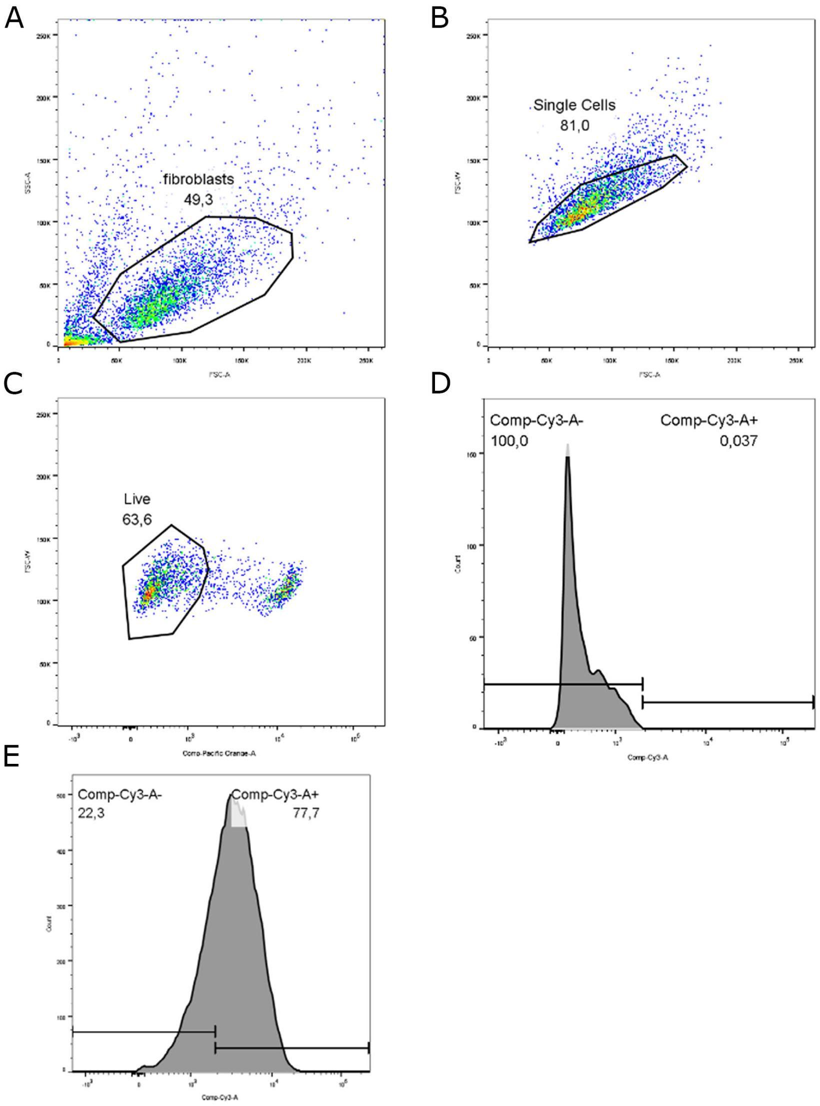

Supplementary Figure 6: Transfection efficacy of CCD-18Co fibroblast cells. Transfection efficacy of CCD-18Co fibroblasts cells were tested using siRNA coupled to Cy-3 (siR-Cy3). Fibroblasts were transfected with 50 nM siR-Cy3 in 6 well plates using RNAiMax reagent. Three days post transfection cells were harvested and analyzed by flow cytometry. First, a gate was set on fibroblast cells based on forward scatter area (FSC-A) and side scatter area (SSC-A) with 50% of all events inside the fibroblast gate **(A)**. Subsequently, doublets were excluded by gating on single cells using FSC area and FSC height with > 90% single cells from all fibroblast events **(B)**. Dead cells were excluded by setting a gate on living cells based on fluorescence intensity of Pacific-Orange. Therefore, heat-killed cells were mixed 1:1 with viable cells and the gate was set on Pacific-Orange negative subset resulting in live subset with 60% of all single cell events **(C)**. Finally, the gate for Cy3 negative and positive cells was set using sample stained with an isotype control resulting in >99% Cy3 negative events **(D)**. Cells transfected with 50 nM siR-Cy3 using RNAiMax transfection reagent showed a viability of 94 % and a transfection efficacy (Cy3 positive events) of 80% which is shown in **(E)**.

Supplementary Table 1: Detailed information about gene- and miRNA expression datasets used for modeling.

See Supplementary File 1

Supplementary Table 2: Detailed information about miRNA transfection datasets used for model validation.

See Supplemenatry File 2

Supplementary Table 3: Analyzed cell-type specific gene- and miRNA expression datasets

ID	Tissue type	Cell type	Method	Data type	Platform	num Samples	Reference
GSE30292	Colon cancer	Tumor	laser micro-dissection	Gene exp.	Affymetrix HG U133 Plus 2.0 MA	3	Christensen
GSE30292	Colon cancer	TAF	primary cell culture	Gene exp.	Affymetrix HG U133 Plus 2.0 MA	3	Christensen
GSE39396	Colon cancer	Epithelial	FACS	Gene exp.	Affymetrix HT HG-U133+ PM Array Plate	6	Calon
GSE39396	Colon cancer	Fibroblast	FACS	Gene exp.	Affymetrix HT HG-U133+ PM Array Plate	6	Calon
GSE35602	Colon cancer	Stroma	laser micro-dissection	Gene exp.	Agilent-014850 WHG MA 4x44K	13	Nishida
GSE35602	Normal colon tissue	Stroma	laser micro-dissection	Gene exp.	Agilent-014850 WHG MA 4x44K	4	Nishida
GSE35602	Colon cancer	Epithelial	laser micro-dissection	Gene exp.	Agilent-014850 WHG MA 4x44K	13	Nishida
GSE35602	Normal colon tissue	Epithelial	laser micro-dissection	Gene exp.	Agilent-014850 WHG MA 4x44K	4	Nishida
GSE35602	Colon cancer	Stroma	laser micro-dissection	MiRNA exp.	Agilent-019118 Human miRNA MA 2.0 G4470B	13	Nishida
GSE35602	Normal colon tissue	Stroma	laser micro-dissection	MiRNA exp.	Agilent-019118 Human miRNA MA 2.0 G4470B	4	Nishida
GSE35602	Colon cancer	Epithelial	laser micro-dissection	MiRNA exp.	Agilent-019118 Human miRNA MA 2.0 G4470B	4	Nishida
GSE35602	Normal colon tissue	Epithelial	laser micro-dissection	MiRNA exp.	Agilent-019118 Human miRNA MA 2.0 G4470B	4	Nishida
E-MTAB-2479	Colon cancer	Tumor	micro-dissection	MiRNA exp.	Agilent G13 human miRNA MA 8x15	55	Scarpati
E-MTAB-2479	Colon cancer	Stroma	micro-dissection	MiRNA exp.	Agilent G13 human miRNA MA 8x15	49	Scarpati

TAF = tumor-associated fibroblasts, MA = microarray, FACS = fluorescence-activated cell scanning, exp. = expression, WHG = Whole Human Genome, HG = Human Genome.

Supplementary Table 4: Predicted miRNA binding sites of ECM target genes

MiRNA	Target gene	MirWalk	Pictar	PITA	RNA_22	TargetScan (Conserved)	TargetScan (non-Conserved)
hsa-mir-17	DST	Yes	Yes	Yes	Yes	Yes	Yes
hsa-mir-17	ETS1	---	---	Yes	Yes	---	Yes
hsa-mir-17	FBN1	---	---	Yes	Yes	---	---
hsa-mir-17	FGF2	Yes	---	Yes	Yes	Yes	Yes
hsa-mir-17	FN1	---	---	Yes	---	---	---
hsa-mir-17	ITGAV	---	---	Yes	Yes	---	---
hsa-mir-17	ITGB1	---	---	Yes	Yes	Yes	---
hsa-mir-17	PXDN	---	---	Yes	Yes	Yes	Yes
hsa-mir-17	TIMP2	---	Yes	Yes	Yes	Yes	---
hsa-mir-192	FN1	---	---	---	Yes	---	---
hsa-mir-192	KDR	---	---	Yes	Yes	---	---
hsa-mir-192	MMP2	---	---	Yes	Yes	---	---
hsa-mir-192	TGFB1	---	---	---	---	Yes	---
hsa-mir-200c	DST	---	---	Yes	Yes	Yes	---
hsa-mir-200c	FGF2	---	---	Yes	Yes	Yes	---
hsa-mir-200c	ITGAV	Yes	Yes	Yes	Yes	Yes	Yes
hsa-mir-200c	ITGB1	---	---	Yes	---	---	Yes
hsa-mir-200c	PLOD1	---	---	---	Yes	---	---
hsa-mir-200c	SPARC	Yes	---	---	Yes	---	---

Tested miRNA - target gene pairs are listed row-wise, used binding site prediction tools represent the columns. A pair was marked as predicted if at least one binding site was found with the respective tool.

Supplementary Table 5: Used gene-specific primer pairs for qPCR experiments.

See Supplementary File 3

Supplementary Table 6: Predicted or validated miRNA binding sites of candidate house-keeping genes.

See Supplementary File 4

Supplementary Table 7: Significance values of enrichment tests in miRNA transfection experiments.

See Supplementary File 5

Supplementary Table 8: Enriched gene sets per miRNA, grouped by colorectal cancer subgroups and expression direction.

See Supplemenatry File 6

Supplementary Table 9: Predicted miRNA - target gene pairs involved in extracellular matrix remodeling, grouped by the molecular function of the target genes.

See Supplementary File 7

Supplementary Table 10: Differentially expressed ECM-related genes.

See Supplementary File 8

Supplementary Table 11: Differentially expressed ECM-related miRNAs.

See Supplementary File 9

Supplementary Table 12: Expression fold changes of the identified candidate genes.

See Supplementary File 10

Supplementary Table 13: Correlation coefficients and significance levels of miRNA expression and protein abundance

Mirna	Target Gene	Correlation coefficient	P-value
hsa-mir-200c	FN1	-0.46	2.94E-04
hsa-mir-17	ITGAV	-0.44	5.08E-04
hsa-mir-17	FBN1	-0.41	1.26E-03
hsa-mir-17	FSCN1	-0.36	3.74E-03
hsa-mir-17	ITGB1	-0.36	4.17E-03
hsa-mir-17	LAMC1	-0.33	7.86E-03
hsa-mir-200c	ITGAV	-0.32	9.03E-03
hsa-mir-200c	ITGB1	-0.30	1.26E-02
hsa-mir-17	MMP2	-0.26	2.60E-02
hsa-mir-192	SPARC	-0.22	4.93E-02
hsa-mir-192	FN1	-0.22	5.49E-02
hsa-mir-192	MMP2	-0.22	5.56E-02
hsa-mir-200c	SERPINH1	-0.21	5.86E-02
hsa-mir-192	FBN1	-0.20	7.47E-02
hsa-mir-192	ITGAV	-0.18	8.96E-02
hsa-mir-192	ITGB1	-0.18	8.95E-02
hsa-mir-17	FN1	-0.16	1.22E-01
hsa-mir-200c	PLOD1	-0.12	2.06E-01
hsa-mir-192	LOXL2	-0.08	2.91E-01
hsa-mir-200c	SPARC	-0.07	3.21E-01
hsa-mir-200c	NCAM1	-0.05	4.01E-01
hsa-mir-17	DST	-0.03	4.51E-01
hsa-mir-200c	DST	-0.02	4.83E-01
hsa-mir-200c	FBLN5	0.00	5.37E-01
hsa-mir-17	PXDN	0.04	6.21E-01
hsa-mir-192	PLOD1	0.05	6.62E-01
hsa-mir-192	PXDN	0.06	6.77E-01
hsa-mir-192	DST	0.08	7.19E-01
