# Supplementary material for: MiR-192, miR-200c and miR-17 are fibroblast-mediated inhibitors of colorectal cancer invasion
Source: Oncotarget. 2018 Oct 30;9(85):35559–80. doi: 10.18632/oncotarget.26263 (PMC6238973; doi:10.18632/oncotarget.26263)
Supplement: Supplementary file 8 [file oncotarget-09-35559-s008.docx]

## **Supplementary Table 9. Predicted miRNA - target gene pairs involved in extracellular matrix remodeling, grouped by the molecular function of the target genes**

| 1. **ECM component** | | | | | | | |
| --- | --- | --- | --- | --- | --- | --- | --- |
| **Mirna** | **Gene** |  | **Mirna** | **Gene** |  | **Mirna** | **Gene** |
| hsa-mir-29b-1 | COL10A1 |  | hsa-mir-29b-1 | FBN1 |  | hsa-mir-16-1 | COL4A2 |
| hsa-mir-26b | COL12A1 |  | hsa-mir-29b-2 | FBN1 |  | hsa-mir-16-2 | COL4A2 |
| hsa-mir-26b | COL15A1 |  | hsa-let-7g | FN1 |  | hsa-mir-26b | COL4A2 |
| hsa-mir-29b-1 | COL15A1 |  | hsa-mir-200c | FN1 |  | hsa-mir-29a | COL4A2 |
| hsa-mir-29b-2 | COL15A1 |  | hsa-mir-26b | FN1 |  | hsa-mir-29b-1 | COL4A2 |
| hsa-mir-29b-1 | COL1A1 |  | hsa-mir-186 | NID1 |  | hsa-mir-29b-2 | COL4A2 |
| hsa-mir-29b-2 | COL1A1 |  | hsa-mir-29b-1 | NID1 |  | hsa-mir-26b | COL5A1 |
| hsa-let-7g | COL1A2 |  | hsa-mir-29b-2 | NID1 |  | hsa-mir-29a | COL5A2 |
| hsa-mir-32 | COL23A1 |  | hsa-mir-32 | FBLN2 |  | hsa-mir-29b-1 | COL5A2 |
| hsa-mir-29a | COL3A1 |  | hsa-mir-548d-1 | FBLN2 |  | hsa-mir-29b-2 | COL5A2 |
| hsa-mir-29b-1 | COL3A1 |  | hsa-mir-128-1 | FBLN5 |  | hsa-let-7g | COL8A1 |
| hsa-mir-29b-2 | COL3A1 |  | hsa-mir-128-2 | FBLN5 |  | hsa-mir-190 | COL8A1 |
| hsa-mir-16-1 | COL4A1 |  | hsa-mir-200c | FBLN5 |  | hsa-mir-93 | LAMA4 |
| hsa-mir-29a | COL4A1 |  | hsa-mir-26b | FBLN5 |  | hsa-mir-16-1 | LAMB1 |
| hsa-mir-29b-1 | COL4A1 |  | hsa-mir-7-1 | FBLN5 |  | hsa-mir-15b | LAMC1 |
| hsa-mir-29b-2 | COL4A1 |  | hsa-mir-32 | SPOCK2 |  | hsa-mir-16-1 | LAMC1 |
| hsa-mir-190 | LAMC1 |  | hsa-mir-192 | FBN1 |  | hsa-mir-16-2 | LAMC1 |
| hsa-mir-93 | LAMC1 |  | hsa-mir-29a | FBN1 |  | hsa-mir-17 | LAMC1 |

| 1. **ECM degradation** | | | | | | | |
| --- | --- | --- | --- | --- | --- | --- | --- |
| **Mirna** | **Gene** |  | **Mirna** | **Gene** |  | **Mirna** | **Gene** |
| hsa-mir-15b | FGF2 |  | hsa-mir-16-2 | CTSD |  | hsa-mir-17 | MMP2 |
| hsa-mir-16-2 | FGF2 |  | hsa-mir-26a-1 | ADAM17 |  | hsa-mir-29a | MMP2 |
| hsa-mir-186 | FGF2 |  | hsa-mir-26b | ADAM17 |  | hsa-mir-29b-1 | MMP2 |
| hsa-mir-192 | FGF2 |  | hsa-mir-128-1 | ADAMTS5 |  | hsa-mir-29b-2 | MMP2 |
| hsa-mir-25 | FGF2 |  | hsa-mir-128-2 | ADAMTS5 |  | hsa-mir-29b-1 | MMP9 |
| hsa-mir-26b | FGF2 |  | hsa-mir-7-1 | ADAMTS5 |  | hsa-mir-29b-2 | MMP9 |
| hsa-mir-32 | FGF2 |  | hsa-mir-190 | MMP16 |  | hsa-mir-15b | ABL2 |
| hsa-mir-7-1 | FGF2 |  | hsa-mir-29b-1 | MMP16 |  |  |  |

| **c) ECM synthesis / integrity maintenance** | | | | | | | |
| --- | --- | --- | --- | --- | --- | --- | --- |
| **Mirna** | **Gene** |  | **Mirna** | **Gene** |  | **Mirna** | **Gene** |
| hsa-mir-15b | CCDC80 |  | hsa-mir-548d-1 | RECK |  | hsa-mir-15b | TLL1 |
| hsa-mir-16-2 | CCDC80 |  | hsa-mir-200c | SERPINH1 |  | hsa-mir-16-1 | TLL1 |
| hsa-mir-186 | CCDC80 |  | hsa-mir-26b | SERPINH1 |  | hsa-mir-16-2 | TLL1 |
| hsa-mir-190 | CCDC80 |  | hsa-mir-29b-1 | SERPINH1 |  | hsa-mir-26b | MATN3 |
| hsa-mir-548d-1 | CCDC80 |  | hsa-mir-192 | SPARC |  | hsa-mir-192 | PLOD1 |
| hsa-mir-29b-1 | LOX |  | hsa-mir-29b-1 | SPARC |  | hsa-mir-26b | PLOD2 |
| hsa-mir-93 | LOXL1 |  | hsa-mir-200c | TIMP2 |  | hsa-mir-15b | RECK |
| hsa-mir-192 | LOXL2 |  | hsa-mir-128-1 | A2M |  | hsa-mir-16-2 | RECK |
| hsa-mir-26a-1 | LOXL2 |  | hsa-mir-128-2 | A2M |  | hsa-mir-190 | RECK |
| hsa-mir-26b | LOXL2 |  | hsa-mir-29b-1 | BMP1 |  | hsa-mir-25 | RECK |
| hsa-mir-29b-1 | LOXL2 |  | hsa-mir-29b-2 | BMP1 |  | hsa-mir-26a-1 | CTGF |
| hsa-mir-128-2 | EFEMP2 |  | hsa-mir-7-1 | CRTAP |  | hsa-mir-128-1 | EFEMP2 |
| hsa-mir-29b-1 | P3H1 |  | hsa-mir-16-2 | CTGF |  |  |  |
