# Supplementary material for: MiR-192, miR-200c and miR-17 are fibroblast-mediated inhibitors of colorectal cancer invasion
Source: Oncotarget. 2018 Oct 30;9(85):35559–80. doi: 10.18632/oncotarget.26263 (PMC6238973; doi:10.18632/oncotarget.26263)
Supplement: Supplementary file 9 [file oncotarget-09-35559-s009.docx]

## **Supplementary Table 10. Differentially expressed ECM-related genes**

**a)** In the dataset of Christensen *et al.* [46] (tumor cells vs. primary cell culture of tumor-associated fibroblasts), significantly differentially expressed genes are marked in red.

|  | **Gene** | **P-value** | **Adj.p-value** | **Test statistic** |
| --- | --- | --- | --- | --- |
| **ECM component** | COL10A1 | 4.80E-01 | 6.66E-01 | -8.53E-01 |
|  | COL12A1 | 1.41E-02 | 1.15E-01 | -6.04E+00 |
|  | COL15A1 | 1.05E-01 | 2.96E-01 | -2.81E+00 |
|  | COL1A1 | 1.25E-03 | 4.77E-02 | -1.87E+01 |
|  | COL1A2 | 9.60E-03 | 9.99E-02 | -8.97E+00 |
|  | COL23A1 | 2.24E-01 | 4.39E-01 | -1.59E+00 |
|  | COL3A1 | 2.46E-03 | 6.13E-02 | -7.10E+00 |
|  | COL4A1 | 2.45E-04 | 2.93E-02 | -1.25E+01 |
|  | COL4A2 | 5.53E-04 | 3.88E-02 | -1.01E+01 |
|  | COL5A1 | 9.63E-04 | 4.42E-02 | -1.33E+01 |
|  | COL5A2 | 3.79E-03 | 7.04E-02 | -1.04E+01 |
|  | COL8A1 | 1.28E-02 | 1.10E-01 | -8.30E+00 |
|  | LAMA4 | 1.09E-01 | 3.02E-01 | -2.58E+00 |
|  | LAMB1 | 4.03E-02 | 1.82E-01 | -4.33E+00 |
|  | LAMC1 | 1.46E-02 | 1.17E-01 | -8.09E+00 |
|  | FBN1 | 3.05E-03 | 6.54E-02 | -9.12E+00 |
|  | FN1 | 2.02E-01 | 4.17E-01 | -1.87E+00 |
|  | NID1 | 1.47E-01 | 3.54E-01 | -2.27E+00 |
|  | FBLN2 | 1.21E-01 | 3.16E-01 | -2.35E+00 |
|  | FBLN5 | 2.35E-02 | 1.44E-01 | -5.72E+00 |
|  | SPOCK2 | 3.54E-01 | 5.63E-01 | 1.12E+00 |
| **ECM degradation** | FGF2 | 1.03E-02 | 1.02E-01 | -9.59E+00 |
|  | CTSD | 4.76E-02 | 1.96E-01 | -2.95E+00 |
|  | ADAM17 | 4.49E-01 | 6.42E-01 | 8.49E-01 |
|  | ADAMTS5 | 5.02E-03 | 7.73E-02 | -1.18E+01 |
|  | MMP16 | 1.20E-01 | 3.15E-01 | -2.61E+00 |
|  | MMP2 | 8.85E-01 | 9.39E-01 | -1.59E-01 |
|  | MMP9 | 7.22E-01 | 8.38E-01 | 4.05E-01 |
|  | ABL2 | 1.90E-04 | 2.76E-02 | -1.33E+01 |
| **Others** | MYH11 | 2.44E-02 | 1.46E-01 | -3.77E+00 |
|  | ACTN1 | 1.09E-03 | 4.52E-02 | -1.20E+01 |
|  | ATP7A | 3.56E-01 | 5.65E-01 | 1.09E+00 |
|  | CRISPLD2 | 2.32E-04 | 2.85E-02 | -2.46E+01 |
|  | SNCA | 4.24E-01 | 6.22E-01 | -9.19E-01 |
|  | CSGALNACT1 | 9.25E-01 | 9.61E-01 | -1.02E-01 |
|  | ETS1 | 6.91E-02 | 2.39E-01 | -3.39E+00 |
|  | PHLDB2 | 1.31E-01 | 3.32E-01 | -2.17E+00 |
|  | FOXC1 | 1.75E-01 | 3.87E-01 | -1.67E+00 |
|  | FOXF2 | 1.56E-03 | 5.25E-02 | -9.85E+00 |
|  | KDR | 3.65E-01 | 5.74E-01 | 1.06E+00 |
|  | LCP1 | 2.93E-02 | 1.57E-01 | 3.77E+00 |
|  | MKX | 1.30E-02 | 1.11E-01 | -8.20E+00 |
|  | NRXN1 | 1.68E-01 | 3.79E-01 | 1.73E+00 |

| **ECM synthesis / integrity maintenance** | CCDC80 | 1.17E-03 | 4.65E-02 | -1.35E+01 |
| --- | --- | --- | --- | --- |
|  | TLL1 | 2.08E-01 | 4.23E-01 | -1.78E+00 |
|  | EFEMP2 | 6.37E-05 | 2.10E-02 | -1.77E+01 |
|  | CTGF | 2.85E-02 | 1.55E-01 | -5.46E+00 |
|  | LOX | 1.75E-04 | 2.72E-02 | -1.61E+01 |
|  | LOXL1 | 1.04E-04 | 2.23E-02 | -2.15E+01 |
|  | LOXL2 | 1.06E-01 | 2.97E-01 | -2.60E+00 |
|  | CRTAP | 4.37E-03 | 7.32E-02 | -7.72E+00 |
|  | BMP1 | 7.55E-02 | 2.50E-01 | -2.49E+00 |
|  | A2M | 2.10E-02 | 1.37E-01 | -3.70E+00 |
|  | MATN3 | 7.97E-02 | 2.57E-01 | -2.59E+00 |
|  | PLOD1 | 9.17E-05 | 2.23E-02 | -1.67E+01 |
|  | PLOD2 | 4.39E-02 | 1.89E-01 | -3.53E+00 |
|  | RECK | 5.46E-03 | 7.91E-02 | -5.47E+00 |
|  | TIMP2 | 9.55E-05 | 2.23E-02 | -1.57E+01 |
|  | SPARC | 3.84E-03 | 7.08E-02 | -8.58E+00 |
|  | SERPINH1 | 1.57E-03 | 5.25E-02 | -1.20E+01 |
| **ECM-cell connection / signaling** | DMD | 1.65E-01 | 3.76E-01 | -1.75E+00 |
|  | ITGA4 | 7.49E-03 | 9.01E-02 | -9.91E+00 |
|  | ITGAV | 1.04E-01 | 2.93E-01 | -2.35E+00 |
|  | ITGAX | 7.19E-01 | 8.37E-01 | 3.97E-01 |
|  | ITGB1 | 5.73E-02 | 2.17E-01 | -3.66E+00 |
|  | ITGB2 | 9.51E-02 | 2.81E-01 | -2.32E+00 |
|  | ITGB3 | 2.78E-03 | 6.37E-02 | -6.70E+00 |
|  | PXDN | 4.19E-04 | 3.40E-02 | -2.08E+01 |
|  | JAM2 | 1.43E-03 | 5.08E-02 | -1.55E+01 |
|  | SDC2 | 2.15E-03 | 5.91E-02 | -9.43E+00 |
|  | SDC3 | 8.46E-02 | 2.64E-01 | -2.42E+00 |
|  | APLP2 | 3.05E-02 | 1.60E-01 | -3.57E+00 |
|  | APP | 5.50E-01 | 7.18E-01 | 6.54E-01 |
|  | CLASP1 | 2.64E-02 | 1.51E-01 | -4.85E+00 |
|  | CYR61 | 9.97E-04 | 4.42E-02 | -1.65E+01 |
|  | DST | 5.99E-01 | 7.52E-01 | 5.91E-01 |
|  | ENG | 3.67E-03 | 6.97E-02 | -6.51E+00 |
|  | FSCN1 | 2.51E-04 | 2.93E-02 | -1.73E+01 |
|  | ICAM1 | 1.63E-01 | 3.73E-01 | -2.03E+00 |
|  | ILK | 2.35E-03 | 6.09E-02 | -7.71E+00 |
|  | MFAP3 | 4.59E-01 | 6.49E-01 | -8.22E-01 |
|  | MFAP5 | 2.86E-02 | 1.55E-01 | -5.65E+00 |
|  | NCAM1 | 1.27E-01 | 3.26E-01 | 2.15E+00 |
|  | PDGFA | 7.06E-01 | 8.28E-01 | 4.07E-01 |
|  | PDGFB | 5.14E-01 | 6.93E-01 | 7.36E-01 |
|  | PLEC | 1.00E-02 | 1.02E-01 | -6.65E+00 |
|  | TGFB1 | 1.88E-02 | 1.31E-01 | -4.22E+00 |
|  | TGFB3 | 1.16E-02 | 1.07E-01 | -6.20E+00 |
|  | TGFBR1 | 1.15E-01 | 3.08E-01 | -2.06E+00 |
|  | THBS1 | 4.08E-04 | 3.34E-02 | -1.74E+01 |
|  | VCAM1 | 3.95E-02 | 1.80E-01 | -4.83E+00 |

**b)** In the dataset of Calon *et al.* [47] (epithelial tumor cells vs. fibroblasts)

|  | **Gene** | **P-value** | **Adj.p-value** | **Test statistic** |
| --- | --- | --- | --- | --- |
| **ECM component** | COL10A1 | 2.12E-03 | 2.84E-02 | -5.442 |
|  | COL12A1 | 3.57E-09 | 4.04E-06 | -22.105 |
|  | COL15A1 | 4.24E-05 | 2.12E-03 | -10.348 |
|  | COL1A1 | 2.34E-09 | 3.06E-06 | -33.887 |
|  | COL1A2 | 8.78E-09 | 7.13E-06 | -17.484 |
|  | COL23A1 | 8.25E-01 | 9.09E-01 | 0.226 |
|  | COL3A1 | 1.95E-11 | 8.30E-08 | -43.970 |
|  | COL4A1 | 2.64E-05 | 1.59E-03 | -10.980 |
|  | COL4A2 | 1.76E-07 | 5.10E-05 | -14.936 |
|  | COL5A1 | 7.84E-09 | 7.04E-06 | -31.468 |
|  | COL5A2 | 1.31E-07 | 3.99E-05 | -23.438 |
|  | COL8A1 | 2.39E-06 | 3.23E-04 | -10.306 |
|  | LAMA4 | 6.47E-08 | 2.56E-05 | -15.820 |
|  | LAMB1 | 1.79E-05 | 1.28E-03 | -7.942 |
|  | LAMC1 | 3.61E-08 | 1.98E-05 | -15.573 |
|  | FBN1 | 3.28E-05 | 1.79E-03 | -9.831 |
|  | FN1 | 3.52E-05 | 1.87E-03 | -9.903 |
|  | NID1 | 2.00E-03 | 2.74E-02 | -5.098 |
|  | FBLN2 | 3.02E-04 | 7.95E-03 | -7.790 |
|  | FBLN5 | 2.06E-04 | 6.09E-03 | -6.757 |
|  | SPOCK2 | 4.74E-02 | 1.89E-01 | 2.292 |
| **ECM degradation** | FGF2 | 8.83E-01 | 9.40E-01 | 0.153 |
|  | CTSD | 1.32E-01 | 3.31E-01 | -1.649 |
|  | ADAM17 | 3.26E-01 | 5.47E-01 | -1.034 |
|  | ADAMTS5 | 7.33E-03 | 6.24E-02 | -3.752 |
|  | MMP16 | 1.88E-01 | 4.04E-01 | -1.439 |
|  | MMP2 | 7.47E-04 | 1.47E-02 | -5.298 |
|  | MMP9 | 1.83E-01 | 3.98E-01 | -1.486 |
|  | ABL2 | 3.26E-02 | 1.54E-01 | -2.499 |
| **Others** | MYH11 | 7.62E-03 | 6.40E-02 | -3.993 |
|  | ACTN1 | 1.04E-03 | 1.81E-02 | -5.303 |
|  | ATP7A | 9.14E-01 | 9.58E-01 | 0.110 |
|  | CRISPLD2 | 4.26E-06 | 4.75E-04 | -13.251 |
|  | SNCA | 4.00E-01 | 6.12E-01 | 0.881 |
|  | CSGALNACT1 | 2.96E-01 | 5.19E-01 | -1.123 |
|  | ETS1 | 3.27E-04 | 8.39E-03 | -6.479 |
|  | PHLDB2 | 1.12E-05 | 9.57E-04 | -8.452 |
|  | FOXC1 | 8.64E-01 | 9.30E-01 | 0.179 |
|  | FOXF2 | 1.58E-02 | 9.99E-02 | -3.094 |
|  | KDR | 4.27E-01 | 6.35E-01 | 0.833 |
|  | LCP1 | 5.03E-01 | 6.96E-01 | 0.695 |
|  | MKX | 6.36E-01 | 7.91E-01 | -0.488 |
|  | NRXN1 | 6.11E-02 | 2.16E-01 | 2.112 |

| **ECM synthesis / integrity maintenance** | CCDC80 | 8.76E-04 | 1.63E-02 | -6.705 |
| --- | --- | --- | --- | --- |
|  | TLL1 | 4.75E-01 | 6.74E-01 | 0.744 |
|  | EFEMP2 | 1.94E-07 | 5.51E-05 | -21.642 |
|  | CTGF | 3.23E-07 | 7.92E-05 | -18.657 |
|  | LOX | 7.69E-04 | 1.50E-02 | -6.022 |
|  | LOXL1 | 1.19E-05 | 9.86E-04 | -8.835 |
|  | LOXL2 | 7.43E-06 | 7.03E-04 | -17.136 |
|  | CRTAP | 1.49E-02 | 9.64E-02 | -3.078 |
|  | BMP1 | 9.05E-03 | 7.14E-02 | -3.272 |
|  | A2M | 6.80E-05 | 2.93E-03 | -6.660 |
|  | MATN3 | 3.21E-02 | 1.53E-01 | -2.499 |
|  | PLOD1 | 6.30E-05 | 2.78E-03 | -6.622 |
|  | PLOD2 | 1.90E-06 | 2.81E-04 | -10.670 |
|  | RECK | 4.46E-01 | 6.51E-01 | 0.798 |
|  | TIMP2 | 1.33E-08 | 9.83E-06 | -18.615 |
|  | SPARC | 3.44E-10 | 5.33E-07 | -28.187 |
|  | SERPINH1 | 6.68E-09 | 6.32E-06 | -18.113 |
| **ECM-cell connection / signaling** | DMD | 4.78E-01 | 6.77E-01 | -0.737 |
|  | ITGA4 | 1.61E-02 | 1.01E-01 | -3.383 |
|  | ITGAV | 3.63E-06 | 4.24E-04 | -9.477 |
|  | ITGAX | 3.17E-01 | 5.38E-01 | 1.055 |
|  | ITGB1 | 8.12E-01 | 9.01E-01 | -0.249 |
|  | ITGB2 | 1.45E-01 | 3.50E-01 | 1.598 |
|  | ITGB3 | 6.45E-03 | 5.78E-02 | -4.056 |
|  | PXDN | 5.59E-08 | 2.44E-05 | -17.158 |
|  | JAM2 | 3.20E-01 | 5.41E-01 | -1.065 |
|  | SDC2 | 6.18E-08 | 2.51E-05 | -14.498 |
|  | SDC3 | 1.79E-03 | 2.55E-02 | -4.219 |
|  | APLP2 | 8.21E-01 | 9.06E-01 | -0.233 |
|  | APP | 1.60E-02 | 1.01E-01 | -3.268 |
|  | CLASP1 | 2.97E-02 | 1.45E-01 | -2.536 |
|  | CYR61 | 6.10E-07 | 1.23E-04 | -19.049 |
|  | DST | 7.13E-05 | 3.00E-03 | -6.489 |
|  | ENG | 7.89E-05 | 3.21E-03 | -6.514 |
|  | FSCN1 | 4.08E-04 | 9.77E-03 | -7.169 |
|  | ICAM1 | 8.47E-07 | 1.54E-04 | -11.670 |
|  | ILK | 9.18E-06 | 8.24E-04 | -9.999 |
|  | MFAP3 | 3.59E-03 | 4.02E-02 | -4.623 |
|  | MFAP5 | 5.58E-02 | 2.07E-01 | -2.458 |
|  | NCAM1 | 9.75E-01 | 9.88E-01 | -0.032 |
|  | PDGFA | 7.58E-02 | 2.42E-01 | -1.986 |
|  | PDGFB | 1.81E-01 | 3.95E-01 | 1.440 |
|  | PLEC | 4.02E-02 | 1.73E-01 | -2.397 |
|  | TGFB1 | 1.27E-04 | 4.22E-03 | -6.190 |
|  | TGFB3 | 7.01E-04 | 1.41E-02 | -4.970 |
|  | TGFBR1 | 1.43E-02 | 9.44E-02 | -3.234 |
|  | THBS1 | 1.97E-06 | 2.86E-04 | -9.831 |
|  | VCAM1 | 1.35E-04 | 4.44E-03 | -6.497 |

**c)** In the dataset of Nishida *et al.* [48] (epithelial cells vs. stroma cells, all samples)

|  | **Gene** | **P-value** | **Adj.p-value** | **Test statistic** |
| --- | --- | --- | --- | --- |
| **ECM component** | COL10A1 | 1.63E-02 | 5.41E-02 | -2.579 |
|  | COL12A1 | 6.39E-05 | 6.40E-04 | -4.755 |
|  | COL15A1 | 7.00E-08 | 2.60E-06 | -7.067 |
|  | COL1A1 | 6.13E-06 | 9.36E-05 | -5.618 |
|  | COL1A2 | 1.02E-07 | 3.44E-06 | -7.375 |
|  | COL23A1 | 1.85E-06 | 3.50E-05 | -5.821 |
|  | COL3A1 | 2.03E-05 | 2.50E-04 | -5.253 |
|  | COL4A1 | 5.71E-06 | 8.86E-05 | -5.619 |
|  | COL4A2 | 4.01E-09 | 2.92E-07 | -8.726 |
|  | COL5A1 | 1.04E-06 | 2.22E-05 | -6.360 |
|  | COL5A2 | 1.65E-06 | 3.19E-05 | -6.297 |
|  | COL8A1 | 9.29E-05 | 8.74E-04 | -4.640 |
|  | LAMA4 | 1.85E-06 | 3.50E-05 | -5.820 |
|  | LAMB1 | 1.55E-04 | 1.32E-03 | -4.289 |
|  | LAMC1 | 3.61E-07 | 9.52E-06 | -6.664 |
|  | FBN1 | 7.98E-06 | 1.16E-04 | -5.556 |
|  | FN1 | 5.42E-02 | 1.35E-01 | -1.998 |
|  | NID1 | 2.29E-06 | 4.17E-05 | -5.743 |
|  | FBLN2 | 7.40E-05 | 7.22E-04 | -4.608 |
|  | FBLN5 | 1.49E-10 | 3.53E-08 | -10.322 |
|  | SPOCK2 | 9.47E-07 | 2.07E-05 | -6.183 |
| **ECM degradation** | FGF2 | 2.18E-07 | 6.32E-06 | -6.831 |
|  | CTSD | 4.75E-01 | 6.48E-01 | -0.723 |
|  | ADAM17 | 1.26E-02 | 4.45E-02 | -2.653 |
|  | ADAMTS5 | 8.09E-10 | 1.02E-07 | -8.807 |
|  | MMP16 | 6.42E-02 | 1.53E-01 | -1.919 |
|  | MMP2 | 9.58E-07 | 2.09E-05 | -6.399 |
|  | MMP9 | 2.05E-10 | 4.12E-08 | -9.129 |
|  | ABL2 | 2.36E-04 | 1.87E-03 | -4.146 |
| **Others** | MYH11 | 1.43E-06 | 2.83E-05 | -5.974 |
|  | ACTN1 | 4.41E-03 | 1.99E-02 | -3.067 |
|  | ATP7A | 4.53E-01 | 6.29E-01 | -0.760 |
|  | CRISPLD2 | 2.78E-07 | 7.77E-06 | -6.554 |
|  | SNCA | 7.95E-06 | 1.16E-04 | -5.326 |
|  | CSGALNACT1 | 1.02E-06 | 2.17E-05 | -6.149 |
|  | ETS1 | 1.09E-08 | 6.06E-07 | -7.664 |
|  | PHLDB2 | 8.05E-08 | 2.88E-06 | -6.920 |
|  | FOXC1 | 4.32E-02 | 1.13E-01 | -2.120 |
|  | FOXF2 | 4.46E-09 | 3.18E-07 | -8.083 |
|  | KDR | 2.90E-08 | 1.27E-06 | -7.379 |
|  | LCP1 | 3.99E-08 | 1.64E-06 | -7.341 |
|  | MKX | 3.79E-04 | 2.73E-03 | -4.134 |
|  | NRXN1 | 5.41E-01 | 7.03E-01 | -0.618 |

| **ECM synthesis / integrity maintenance** | CCDC80 | 6.17E-07 | 1.47E-05 | -6.228 |
| --- | --- | --- | --- | --- |
|  | TLL1 | 6.88E-01 | 8.07E-01 | -0.405 |
|  | EFEMP2 | 3.11E-09 | 2.50E-07 | -8.437 |
|  | CTGF | 2.30E-05 | 2.76E-04 | -5.134 |
|  | LOX | 7.54E-05 | 7.34E-04 | -4.577 |
|  | LOXL1 | 3.22E-07 | 8.77E-06 | -7.153 |
|  | LOXL2 | 2.89E-10 | 5.10E-08 | -9.774 |
|  | CRTAP | 4.99E-02 | 1.26E-01 | -2.042 |
|  | BMP1 | 3.88E-03 | 1.81E-02 | -3.224 |
|  | A2M | 4.55E-08 | 1.82E-06 | -7.273 |
|  | MATN3 | 1.86E-02 | 5.99E-02 | -2.525 |
|  | PLOD1 | 1.07E-01 | 2.24E-01 | -1.659 |
|  | PLOD2 | 1.20E-02 | 4.29E-02 | -2.664 |
|  | RECK | 9.08E-07 | 2.01E-05 | -6.204 |
|  | TIMP2 | 6.60E-08 | 2.47E-06 | -7.159 |
|  | SPARC | 1.34E-07 | 4.33E-06 | -7.187 |
|  | SERPINH1 | 2.12E-03 | 1.12E-02 | -3.380 |
| **ECM-cell connection / signaling** | DMD | 9.72E-02 | 2.09E-01 | -1.710 |
|  | ITGA4 | 1.89E-08 | 9.07E-07 | -8.298 |
|  | ITGAV | 8.17E-03 | 3.19E-02 | -2.852 |
|  | ITGAX | 1.22E-06 | 2.51E-05 | -6.317 |
|  | ITGB1 | 1.39E-01 | 2.71E-01 | -1.535 |
|  | ITGB2 | 5.42E-08 | 2.07E-06 | -7.289 |
|  | ITGB3 | 3.13E-07 | 8.55E-06 | -6.441 |
|  | PXDN | 2.58E-08 | 1.17E-06 | -7.503 |
|  | JAM2 | 3.50E-08 | 1.46E-06 | -7.386 |
|  | SDC2 | 9.39E-07 | 2.06E-05 | -6.123 |
|  | SDC3 | 1.95E-02 | 6.18E-02 | -2.477 |
|  | APLP2 | 2.92E-02 | 8.41E-02 | 2.310 |
|  | APP | 2.29E-02 | 7.00E-02 | 2.430 |
|  | CLASP1 | 6.78E-04 | 4.44E-03 | -3.862 |
|  | CYR61 | 8.72E-10 | 1.08E-07 | -8.572 |
|  | DST | 7.40E-01 | 8.43E-01 | -0.334 |
|  | ENG | 6.54E-09 | 4.25E-07 | -8.522 |
|  | FSCN1 | 4.82E-07 | 1.20E-05 | -6.292 |
|  | ICAM1 | 9.52E-09 | 5.49E-07 | -7.698 |
|  | ILK | 3.10E-05 | 3.48E-04 | -4.853 |
|  | MFAP3 | 9.96E-01 | 9.98E-01 | 0.005 |
|  | MFAP5 | 5.43E-04 | 3.70E-03 | -3.956 |
|  | NCAM1 | 1.91E-01 | 3.44E-01 | -1.335 |
|  | PDGFA | 2.53E-01 | 4.19E-01 | -1.171 |
|  | PDGFB | 2.13E-10 | 4.15E-08 | -9.146 |
|  | PLEC | 9.83E-01 | 9.90E-01 | 0.021 |
|  | TGFB1 | 5.86E-10 | 8.06E-08 | -8.863 |
|  | TGFB3 | 1.04E-05 | 1.45E-04 | -5.339 |
|  | TGFBR1 | 1.22E-02 | 4.33E-02 | -2.678 |
|  | THBS1 | 3.30E-04 | 2.44E-03 | -4.043 |
|  | VCAM1 | 1.70E-07 | 5.23E-06 | -6.646 |
