# Supplementary material for: MiR-192, miR-200c and miR-17 are fibroblast-mediated inhibitors of colorectal cancer invasion
Source: Oncotarget. 2018 Oct 30;9(85):35559–80. doi: 10.18632/oncotarget.26263 (PMC6238973; doi:10.18632/oncotarget.26263)
Supplement: Supplementary file 10 [file oncotarget-09-35559-s010.docx]

## **Supplementary Table 11. Differentially expressed ECM-related miRNAs**

**a)** In the dataset of Nishida *et al.* [48] (epithelial cells vs. stroma cells, only tumor samples), significantly differentially expressed miRNAs are marked in red.

| **mirna (TCGA)*** | **mirna (Nishida)*** | **P-value** | **Adj.P-value** | **Test statistic** |
| --- | --- | --- | --- | --- |
| hsa-let-7g | hsa-let-7g | 6.72E-01 | 8.26E-01 | 0.452 |
| hsa-let-7g | hsa-let-7g* | 8.64E-01 | 9.31E-01 | 0.181 |
| hsa-mir-128-1 | hsa-miR-128 | 1.67E-01 | 4.64E-01 | 1.565 |
| hsa-mir-128-2 | hsa-miR-128 | 1.67E-01 | 4.64E-01 | 1.565 |
| hsa-mir-15b | hsa-miR-15b | 1.90E-01 | 4.95E-01 | 1.527 |
| hsa-mir-15b | hsa-miR-15b* | 3.07E-01 | 5.97E-01 | 1.216 |
| hsa-mir-16-1 | hsa-miR-16-1* | 2.21E-01 | 5.15E-01 | -1.290 |
| hsa-mir-16-2 | hsa-miR-16-2* | 4.03E-01 | 6.19E-01 | 0.915 |
| hsa-mir-17 | hsa-miR-17 | 5.40E-03 | 4.16E-02 | 4.198 |
| hsa-mir-17 | hsa-miR-17* | 1.24E-04 | 5.51E-03 | 5.596 |
| hsa-mir-186 | hsa-miR-186 | 3.42E-01 | 6.01E-01 | -1.066 |
| hsa-mir-190 | hsa-miR-190 | 4.13E-01 | 6.22E-01 | 0.933 |
| hsa-mir-192 | hsa-miR-192 | 1.20E-03 | 1.63E-02 | 4.703 |
| hsa-mir-192 | hsa-miR-192* | 9.46E-04 | 1.44E-02 | 5.218 |
| hsa-mir-200c | hsa-miR-200c | 1.96E-02 | 1.18E-01 | 3.322 |
| hsa-mir-200c | hsa-miR-200c* | 2.59E-01 | 5.60E-01 | 1.365 |
| hsa-mir-25 | hsa-miR-25 | 2.00E-01 | 5.03E-01 | 1.471 |
| hsa-mir-26a-1 | hsa-miR-26a | 8.28E-01 | 9.14E-01 | -0.227 |
| hsa-mir-26b | hsa-miR-26b | 7.31E-01 | 8.53E-01 | -0.365 |
| hsa-mir-26b | hsa-miR-26b* | 3.91E-01 | 6.13E-01 | 1.000 |
| hsa-mir-29a | hsa-miR-29a | 3.00E-02 | 1.55E-01 | 2.813 |
| hsa-mir-29a | hsa-miR-29a* | 2.37E-01 | 5.28E-01 | 1.402 |
| hsa-mir-29b-1 | hsa-miR-29b-1* | 1.50E-01 | 4.32E-01 | 1.831 |
| hsa-mir-29b-2 | NA | NA | NA | NA |
| hsa-mir-30c-1 | hsa-miR-30c-1* | 3.33E-01 | 5.97E-01 | 1.110 |
| hsa-mir-32 | hsa-miR-32 | 9.45E-02 | 3.38E-01 | 1.969 |
| hsa-mir-32 | hsa-miR-32* | 8.47E-01 | 9.22E-01 | 0.204 |
| hsa-mir-548d-1 | hsa-miR-548d-3p | 7.01E-01 | 8.40E-01 | 0.409 |
| hsa-mir-548d-1 | hsa-miR-548d-5p | 8.31E-01 | 9.14E-01 | -0.225 |
| hsa-mir-7-1 | hsa-miR-7-1* | 2.27E-01 | 5.19E-01 | 1.449 |
| hsa-mir-93 | hsa-miR-93 | 6.22E-02 | 2.48E-01 | 2.395 |

**b)** In the dataset of Scarpati *et al.* [49] (tumor cells vs stroma cells)

| **mirna (TCGA)*** | **mirna (Scarpati)*** | **P-value** | **Adj.P-value** | **Test statistic** |
| --- | --- | --- | --- | --- |
| hsa-let-7g | hsa-let-7g-5p | 3.33E-01 | 4.24E-01 | -0.973 |
| hsa-mir-128-1 | NA | NA | NA | NA |
| hsa-mir-128-2 | NA | NA | NA | NA |
| hsa-mir-15b | hsa-miR-15b-5p | 9.08E-01 | 9.23E-01 | 0.115 |
| hsa-mir-15b | NA | NA | NA | NA |
| hsa-mir-16-1 | hsa-miR-16-5p | 9.09E-01 | 9.23E-01 | -0.115 |
| hsa-mir-16-2 | hsa-miR-16-5p | 9.09E-01 | 9.23E-01 | -0.115 |
| hsa-mir-17 | hsa-miR-17-5p | 3.06E-03 | 1.41E-02 | 3.034 |
| hsa-mir-186 | NA | NA | NA | NA |
| hsa-mir-190 | NA | NA | NA | NA |
| hsa-mir-192 | hsa-miR-192-5p | 2.28E-04 | 2.22E-03 | 3.838 |
| hsa-mir-200c | hsa-miR-200c-3p | 2.18E-07 | 1.40E-05 | 5.655 |
| hsa-mir-25 | hsa-miR-25-3p | 4.54E-02 | 9.58E-02 | 2.026 |
| hsa-mir-26a-1 | hsa-miR-26a-5p | 1.16E-01 | 1.92E-01 | -1.584 |
| hsa-mir-26b | hsa-miR-26b-5p | 7.25E-02 | 1.35E-01 | -1.815 |
| hsa-mir-29a | hsa-miR-29a-3p | 1.31E-01 | 2.09E-01 | 1.523 |
| hsa-mir-29b-1 | hsa-miR-29b-3p | 3.25E-01 | 4.17E-01 | 0.989 |
| hsa-mir-29b-2 | hsa-miR-29b-3p | 3.25E-01 | 4.17E-01 | 0.989 |
| hsa-mir-30c-1 | hsa-miR-30c-1-3p | 9.55E-01 | 9.67E-01 | -0.056 |
| hsa-mir-32 | NA | NA | NA | NA |
| hsa-mir-548d-1 | NA | NA | NA | NA |
| hsa-mir-7-1 | NA | NA | NA | NA |
| hsa-mir-93 | hsa-miR-93-5p | 1.03E-02 | 3.11E-02 | 2.616 |

* Pre-miRNA identifiers from TCGA were mapped to mature miRNA identifiers used in the respective dataset according to miRBase [50].
