# Supplementary material for: MiR-192, miR-200c and miR-17 are fibroblast-mediated inhibitors of colorectal cancer invasion
Source: Oncotarget. 2018 Oct 30;9(85):35559–80. doi: 10.18632/oncotarget.26263 (PMC6238973; doi:10.18632/oncotarget.26263)
Supplement: Supplementary file 11 [file oncotarget-09-35559-s011.docx]

## **Supplementary Table 12. Expression fold changes of the identified candidate genes**

**a**) After miR-192 transfection with TBP as control gene

| **Gene symbol** | **Log2 Fold Change** |
| --- | --- |
| SPARC | -0.39 |
| FGF2 | -1.55 |
| DST | -0.12 |
| PLOD1 | -0.95 |
| LOXL2 | -1.24 |
| ITGB1 | -1.09 |
| ITGAV | -0.71 |
| FN1 | -0.68 |
| PXDN | -0.33 |
| MMP2 | 0.66 |
| TGFB1 | -0.26 |
| KDR | -0.62 |

**b**) After miR-17 transfection with TBP as control gene

| **Gene symbol** | **Log2 Fold Change** |
| --- | --- |
| TGFB1 | -0.92 |
| FSCN1 | 0.09 |
| LAMC1 | -0.93 |
| MMP2 | -1.64 |
| ITGAV | -0.64 |
| FBN1 | 0.76 |
| PXDN | -0.68 |
| FN1 | -1.53 |
| TIMP2 | -1.51 |
| FGF2 | -0.70 |
| ITGB1 | -1.25 |
| ETS1 | 0.14 |
| DST | -1.07 |

**c**) After miR-200c transfection with UBC as control gene

| **Gene symbol** | **Log2 Fold Change** |
| --- | --- |
| KDR | 0.29 |
| ETS1 | -0.99 |
| TIMP2 | -1.23 |
| NCAM1 | -1.13 |
| SERPINH1 | -1.27 |
| ITGAV | -0.46 |
| ITGB1 | -0.36 |
| FGF2 | -1.69 |
| SPARC | -0.48 |
| FBLN5 | -0.53 |
| FN1 | -1.92 |
| PLOD1 | -0.79 |
| DST | -1.45 |
